# Cold-Triggered Induction of ROS- and Raffinose Metabolism in Freezing-Sensitive Taproot Tissue of Sugar Beet

**DOI:** 10.3389/fpls.2021.715767

**Published:** 2021-09-03

**Authors:** Isabel Keller, Christina Müdsam, C. Martins Rodrigues, Dominik Kischka, Wolfgang Zierer, Uwe Sonnewald, Karsten Harms, Olaf Czarnecki, Karin Fiedler-Wiechers, Wolfgang Koch, H. Ekkehard Neuhaus, Frank Ludewig, Benjamin Pommerrenig

**Affiliations:** ^1^Department of Plant Physiology, University of Kaiserslautern, Kaiserslautern, Germany; ^2^Department of Biochemistry, FAU Erlangen-Nürnberg, Erlangen, Germany; ^3^CRDS, Südzucker AG, Obrigheim/Pfalz, Germany; ^4^KWS SAAT SE & Co. KGaA, Einbeck, Germany

**Keywords:** sugar beet, freezing, pith, reactive oxygen species, raffinose

## Abstract

Sugar beet (*Beta vulgaris* subsp. *vulgaris*) is the exclusive source of sugar in the form of sucrose in temperate climate zones. Sugar beet is grown there as an annual crop from spring to autumn because of the damaging effect of freezing temperatures to taproot tissue. A collection of hybrid and non-hybrid sugar beet cultivars was tested for winter survival rates and freezing tolerance. Three genotypes with either low or high winter survival rates were selected for detailed study of their response to frost. These genotypes differed in the severity of frost injury in a defined inner region in the upper part of the taproot, the so-called pith. We aimed to elucidate genotype- and tissue-dependent molecular processes during freezing and combined analyses of sugar beet anatomy and physiology with transcriptomic and metabolite profiles of leaf and taproot tissues at low temperatures. Freezing temperatures induced strong downregulation of photosynthesis in leaves, generation of reactive oxygen species (ROS), and ROS-related gene expression in taproots. Simultaneously, expression of genes involved in raffinose metabolism, as well as concentrations of raffinose and its intermediates, increased markedly in both leaf and taproot tissue at low temperatures. The accumulation of raffinose in the pith tissue correlated with freezing tolerance of the three genotypes. We discuss a protective role for raffinose and its precursors against freezing damage of sugar beet taproot tissue.

## Introduction

In temperate climate zones (Europe and North America), sugar beet (*Beta vulgaris subsp. vulgaris*) is the exclusive source of sugar (sucrose) for the food industry and a source for bio-energy generation. Sugar beet taproots are able to accumulate sucrose up to nearly 20% of their fresh weight at maturity (Dohm et al., 2013) and provide about 30% of the total sugar produced worldwide (Zhang et al., 2017). Owing to its biennial lifestyle, the plant forms the huge sucrose-storing taproot during the first year of its life cycle. The stored sugar is used to fuel the outgrowth of a flowering seed stalk in the reproductive phase in the second year of growth (Chen et al., 2016). Induction of flowering, however, requires a prolonged exposure to cold (between 5 and 20 weeks at 4–15°C), known as vernalization (Abo-Elwafa et al., 2006; Kockelmann et al., 2010). During vernalization, shoots and taproots prepare for metabolic and functional rearrangements resulting in a switch of their source and sink identities, where shoot metabolism depends on carbon supply from the taproot to allow outgrowth of the seed stalk (Rodrigues et al., 2020). Successful vernalization of sugar beet plants can only occur at low, but above-zero temperatures. This is because, despite the high accumulation of sugars, which are known to stabilize membranes and protect against freezing-induced damages (Anchordoguy et al., 1987; Pommerrenig et al., 2018), sugar beet is sensitive to subzero temperatures (Barbier et al., 1982; Loel and Hoffmann, 2015). This sensitivity limits cultivation of the crop to regions with a vegetative period from spring to late autumn. The limited growth period and the slow formation of leaves in spring are its main yield-limiting factors (Milford and Riley, 1980; Jaggard et al., 2009). Calculations taking an increased freezing and bolting tolerance into account suggested that an elongated growth period of sugar beet might result in an increase of the total sugar yield by about 25% (Hoffmann and Kluge-Severin, 2011). However, overwintering sugar beet plants must be able to withstand freezing temperatures, and therefore, freezing tolerance has become a desirable trait for sugar beet breeders. Freezing temperatures frequently lead to severe yield losses of different crops (Barbier et al., 1982; Fennell, 2004; Maqbool et al., 2010; Chang et al., 2014), as intra- and extracellular ice formation damages plant tissue by rupturing cell membranes or because of cellular dehydration (Burke et al., 1976; Wolfe and Bryant, 1999). On the macroscopic level, the impact of freezing on sugar beet taproot tissue is drastic and results in lethal damage to internal tissue and ultimately, the entire plant. Ice formation and thawing leads to cell rupture and leakage of root sap, which ultimately attracts microorganisms and leads to rot of the taproot (Barbier et al., 1982).

Factors protecting plants against freezing may be soluble sugars, amino acids, or derivatives thereof, which function as osmolytes and can thus lower the freezing point, or prevent cellular dehydration upon freezing. Additionally, some of these metabolites can stabilize enzymes, membranes, and other cellular components (Guy, 1990; Yadav, 2010). In particular, carbohydrates, like fructans or raffinose family oligosaccharides (RFOs), have superior membrane protective abilities and additionally represent potent quenchers of reactive oxygen species (ROS; Pontis, 1989; De Roover et al., 2000; Hincha et al., 2007). Accumulation of high levels of fructans in plants of the Asteraceae familiy, like chicory (*Cichorium intybus*) or Jerusalem artichoke (*Helianthus tuberosus*), for example, renders these species tolerant to freezing damage. Sugar beet does not produce fructan but can synthesize raffinose which is derived from sucrose, especially during storage of taproots (Wyse and Dexter, 1971; Haagenson et al., 2008). However, raffinose biosynthesis is attended by reduction of sucrose levels. In fact, as part of the so-called molasses, raffinose is considered an unwanted contaminant lowering the maximum amount of sucrose extractable from the pulp during industrial processing (Wyse and Dexter, 1971; Ganter et al., 1988). On the other hand, raffinose is important for plant frost tolerance (Pennycooke et al., 2003; Nishizawa et al., 2008; Peters and Keller, 2009).

Raffinose is a trisaccharide consisting of sucrose and galactose and is synthesized *via* a subsequent transfer of activated galactose moieties, donated by galactinol, to sucrose (Sengupta et al., 2015). Two specific enzymes, galactinol synthase (GOLS) and raffinose synthase (RS), mediate the synthesis of galactinol and raffinose, respectively. Galactinol synthase mediates the first metabolic step in raffinose biosynthesis, the conversion of UDP-galactose and myo-inositol to galactinol. Raffinose biosynthesis is specifically upregulated during cold acclimation, and several GOLS genes (*GOLS*) are transcriptionally regulated in *Arabidopsis thaliana via* low temperature response transcription factors of the C-repeat binding factor (CBF) family (Gilmour et al., 1998; Shinwari et al., 1998; Fowler and Thomashow, 2002; Taji et al., 2002). C-repeat binding factor proteins directly regulate cold-responsive (COR) genes, which facilitate the acquisition of cold acclimation and sustained freezing tolerance, as products of COR genes are involved in processes, like the synthesis of osmo-protectants and also raffinose, lipid metabolism, or cell wall modification (Thomashow, 1999; Liu et al., 2019). In sugar beet, CBFs, like CBF3, were upregulated upon cold and freezing temperatures but were only detected in roots (Moliterni et al., 2015). Our study aimed to identify potential metabolic factors protecting the sugar beet against adverse effects of freezing. Such factors would then be potential markers for screening of sugar beet cultivars for high winter hardiness or represent targets for biotechnological and breeding approaches. To identify such targets, transcriptomic and metabolomic analyses of leaf and taproot tissues from sugar beet cultivars differing in their frost tolerance were performed. We found that low temperatures affect the accumulation of diverse sugar and RFO species in different sugar beet tissues and genotypes. In particular, the biosynthesis of inositol and raffinose was spatially regulated as a function of temperature. Finally, we discuss how these compounds might play a direct role in sugar beet frost protection.

## Materials and Methods

### Plant Material and Growth Conditions

Three hybrid sugar beet genotypes with contrasting winter-hardiness phenotypes (GT1, GT2, and GT3; KWS SAAT SE, Germany) were selected from field trials performed in Eastern Europe under continental climate conditions in 2012 and 2013. In sugar beet, such hybrids consist of a F1-mother, consisting of two lines derived from a maternal hybrid pool crossed to a father line being part of an opposite hybrid pool. In field experiments, 100 plants of one genotype were grown per plot applying an alpha lattice design with two replicates per location. For growth analysis under controlled conditions in growth chambers and greenhouse, plants were grown in pots (18×18×25cm) on standard soil substrate ED73 (Einheitserdewerke Patzer, Germany) with 10% (v/v) sand under a 10h light/14h dark cycle. Light intensities were 320μmolm^−2^ s^−1^ and 150μmolm^−2^ s^−1^ for greenhouse and growth chamber experiments, respectively. The light intensities were measured from the soil level.

**Figure 1:**
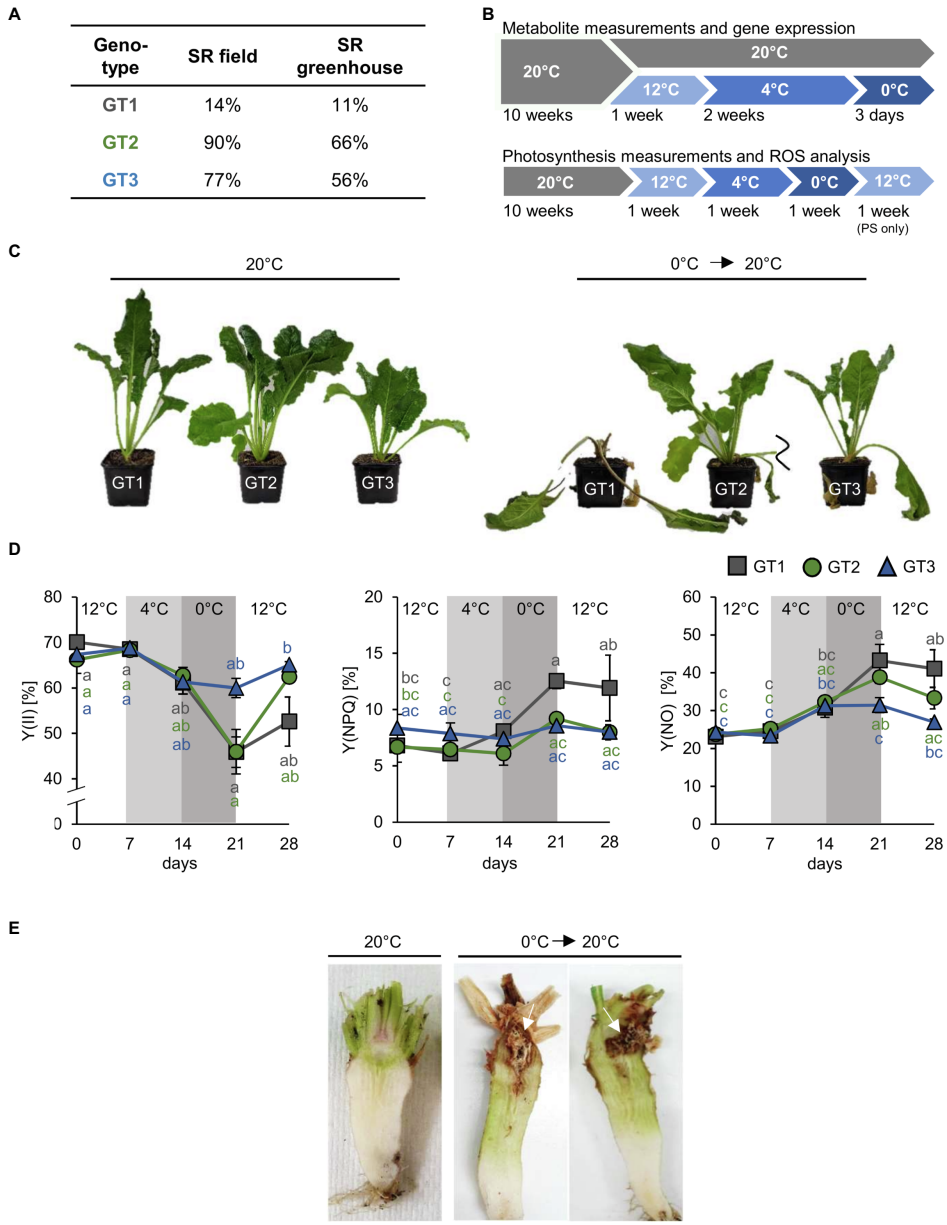
Responses of sugar beet genotypes to freezing temperatures. **(A)** Freezing survival rates (SR) of three analyzed sugar beet cultivars GT1, GT2, and GT3 measured under field (*n=* 100) and greenhouse (*n*=50) conditions given in percent of survived plants. **(B)** Experimental setup for metabolite and photosynthesis measurements. Plants were grown at 20°C for 10weeks. Subsequently, plants were kept at 20°C (control) or transferred to low temperature acclimation conditions (12°C and 4°C). After 4°C treatment, air temperature in the growth chamber was lowered and plants were harvested as the soil temperature was 0°C or below. **(C)** Typical appearance of sugar beet plants of GT1, GT2, and GT3 before and after freezing treatment. **(D)** PAM measurements were performed on leaves of the three different genotypes under cold and freezing conditions, as well as during a post-freezing recovery phase at 12°C. For this, air temperature was kept at 0°C. Quantum yield of photosynthesis [Y(II)] and non-photochemical quenching [Y(NPQ)] and non-regulated energy dissipation [Y(NO)] were measured. Temperature intervals are highlighted in white (12°C), light grey (4°C), or grey (0°C). Values represent the mean of three biological replicates. Error bars represent the standard error of the corresponding mean. The same level of significance, calculated *via* two-way ANOVA with *post-hoc* Tukey HSD test with p<0.05, is given in grey letters for FT1, green letters for GT2, and blue letters for GT3. **(E)** Morphology of sugar beet taproots of genotype GT1 after freezing recovery. Plants were grown at 20°C for 13weeks and afterward were shifted to 12°C, 4°C and 0°C and were retransferred to 20°C for 2 weeks after freezing treatment.

For analysis of gene expression and metabolite concentrations, plants were grown at 20°C for 10weeks, plant population was split, and one group was transferred to 12°C for 1week and 4°C for 2weeks afterward. After 4°C treatment, plants were transferred to-6°C air temperature and were kept there until soil temperature reached 0°C and soil surrounding the taproot was solidly frozen, which took approximately 3days (Figure 1B). Thereby, soil temperature was measured with a Tinytag Plus 2 – TGP-4020 soil temperature data logger (Gemini data loggers, West Sussex, United Kingdom). Control plants were kept at 20°C. Plant tissue was harvested right before start of the temperature treatment under control conditions, after 7day at 12°C, after 14days at 4°C and after 3days at -6°C air/0°C root temperature.

For measurement of photosynthetic parameter, antioxidant concentrations and ROS staining plants were grown at 20°C for 10weeks. After 10weeks, plants were transferred to 12°C and 4°C afterward for 1week each. After 4°C treatment, plants were transferred to 0°C, where they were incubated for 1week. For analysis of photosynthesis, plants were transferred to 12°C for 1week after the treatment at 0°C for recovery (Figure 1B). Plants were harvested under control conditions, after 7days at 12°C, 7days at 4°C, 7days at 0°C and for photosynthesis measurement additionally after 7days at recovery at 12°C.

**Figure 2:**
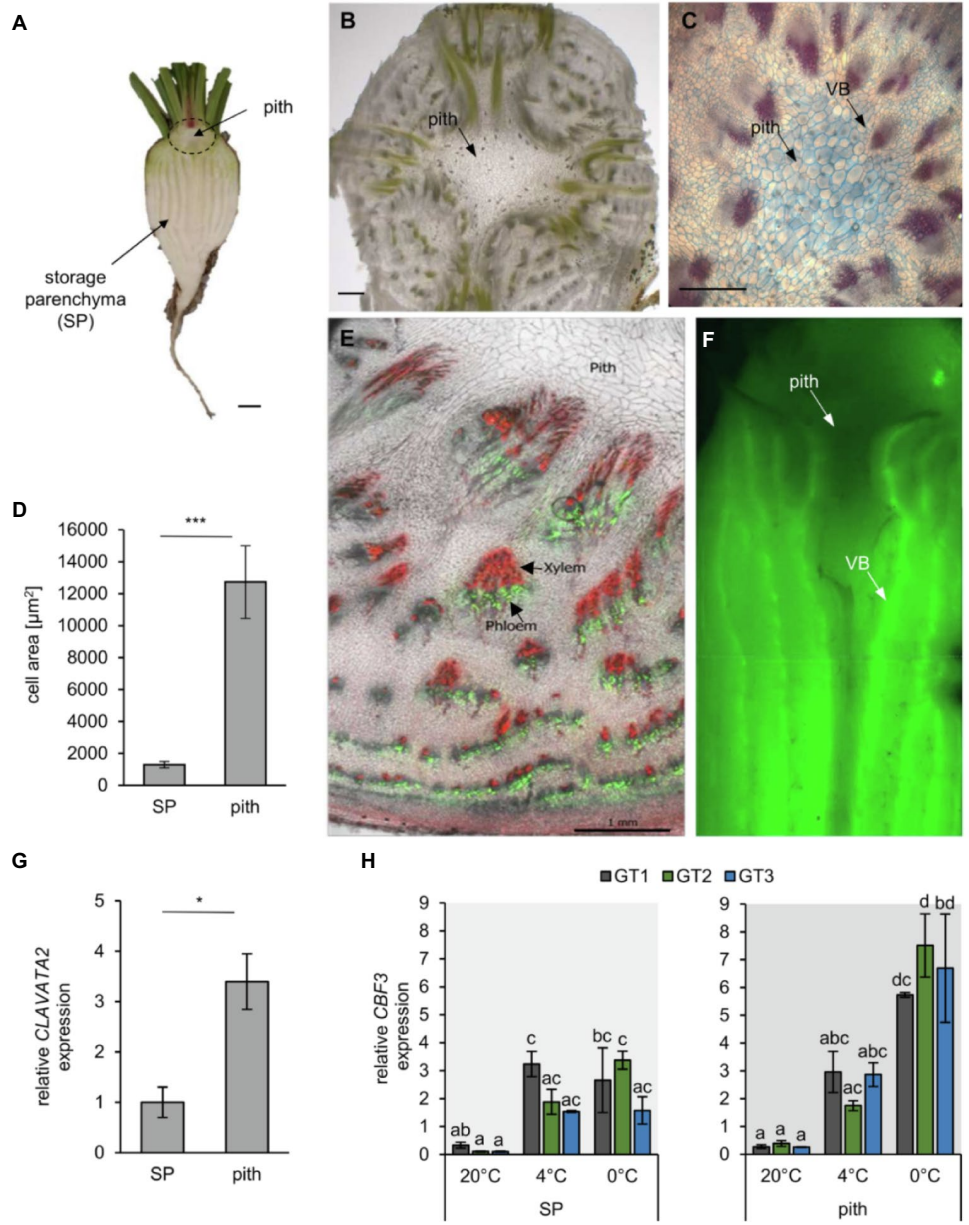
Morphology of the *B. vulgaris* pith tissue. **(A)** longitudinal section of a young taproot. Scale bar representing 1cm. **(B)** Cross section of the pith tissue to reveal its cellular architecture. Scale bar representing 1mm. **(C)** FCA staining of a transverse section of the beet hypocotyl, including the central pith. Lignified xylem cells are stained red, while cellulosic cell walls are stained in blue. Scale bar representing 0.5mm. **(D)** Mean cell area of storage parenchyma (SP) and pith cells measured in sugar beet cross sections using ImageJ. Error bars represent the standard error from seven sections from seven independent taproots. **(E)** Radial phloem-unloading of the fluorescent dye carboxyfluorescein (green). Cell walls were stained with propidium iodine (red), which particularly labels the thick, lignified vessels of the xylem. Scale bar represents 1mm. **(F)** Carboxyfluorescein unloading and distribution from phloem bundles in the sugar beet taproot. Arrows mark the pith tissue or vascular bundles (VB). **(G)** Pith and storage parenchyma were separated, and expression of the shoot meristematic marker *CLAVATA2 was* analyzed. Error bars represent the standard error over three biological replicates. Asterisks indicate significant differences between pith and storage parenchyma [Student’s *t*-test with ^*^p<0.05; ^***^p<0.001 for (D) and (G)] with *n=* 7 in D and *n*=3 in G. **(H)** Expression of the cold-induced transcription factor *CBF3* in SP and pith tissue of the three analyzed genotypes. Error bars represent the standard error over three biological replicates. Letters indicate the same level of significance within genotypes and temperatures, calculated *via* two-way ANOVA with *post-hoc* Tukey HSD test with *p*<0.05.

For all time points, plant tissue was harvested during mid-day at 4h after onset of light. During harvest, plants were dissected into leaf, pith, and storage parenchyma, using a kitchen knife. Pith tissue was identified visually from beets halved lengthwise by its cellular morphology (Figures 1E, 2). Tissue from two plants was pooled and treated as one independent replicate, with a total of four replicates (for metabolite analysis) and three replicates (for ROS and gene expression analysis) per genotype and condition. Harvesting procedure took about 2min per replicate, and therefore, taproot tissue did not thaw during harvest. Harvested material was immediately transferred to liquid nitrogen and samples subjected for metabolite measurements were lyophilized in an Alpha 2–4 LD plus freeze-drier (Christ, Osterode am Harz, Germany). Material was pulverized using a Retsch MM301 mill (Retsch, Haan, Germany) afterward.

### Survival Rate Testing

For survival rate testing, greenhouse and field experiments were carried out. For field experiments, genotypes were sown 3months before start of the frost period at different locations under European continental climate in 2014 and 2015 as described above. Survival rates of genotypes grown under field conditions were only calculated if soil temperatures were recorded below the freezing point. Survival rates are given as mean values of at least two replications in independent years and at different locations. For greenhouse trials, plants were grown in randomized block design with 50 single-plant replications per genotype at an air temperature of-6°C. At least 2weeks after frost treatment, dead or rotten sugar beets plants were counted and survival rate was calculated.

### Chlorophyll Fluorescence Measurements

Photosynthetic activity of three individual plants per genotype was measured using an Imaging-PAM *M-Series*-System (Heinz Walz, Effeltrich, Germany). Plants were placed in the dark for 8min to deplete the energy of PSII. For depletion of energy of PSII, a pretest was performed. Eight min was sufficient to deplete energy of PSII, because Fv/Fmax ratio was greater than 0.75, a value considered as threshold for unstressed plants (Bolhar-Nordenkampf et al., 1989). Capacity of PSII was then measured by saturation with 14cycles of photosynthetically active radiation (76μmol photons m^−2^ s^−1^) light pulses at 0, 50, and 70s. Recorded fluorescence was used for calculation of effective quantum yield of PSII [Y(II)], quantum yield of nonphotochemical quenching [Y(NPQ)], and non-regulated energy dissipation [Y(NO)]. Light curves were recorded by incrementally increasing light pulses with an intensity from PAR 0 (μmol photons m-2s-1) to PAR 726 in 14 steps.

### RNA-Seq Analysis

For RNA-Seq analysis, plant cultivation, RNA extraction, and analysis of RNA-Seq results, including statistical analysis, were performed as described in Martins Rodrigues et al. (2020), and data were made publicly available as BioProject PRJNA602804. RNA was isolated from three biological replicates for each genotype and tissue (leaf and taproot). 100mg of pulverized material was extracted with 1.5ml QIAzol Lysis reagent (Qiagen, Hilden, Germany) and centrifuged at 4°C for 10min at 12,000g. Supernatants were extracted with 300μl chloroform. RNA was precipitated with 750μl isopropanol from aqueous supernatants, after centrifugation 15min 12,000g 4°C. Pellets were washed with 75% EtOH, dried, and resuspended in DEPC-H_2_O. RNA was further purified using the RNeasy KIT (Qiagen, Hilden, Germany). The RNA was quantified (NanoDrop 2000/2000c, Thermo Fisher) prior to further processing, and quality was confirmed using an Agilent Technologies 2,100 Bioanalyzer (Palo Alto, CA, United States). 2μg RNA was transcribed to cDNA. Library preparation and sequencing were done as a custom service by GATC GmbH (Konstanz, Germany). Strand-specific cDNA library was created by isolation of poly(A) + RNA, mRNA fragmentation, followed by random primed cDNA synthesis. Paired-end sequencing was performed using Illumina HiSeq with 2x150bp read length. Sequencing results were provided in FASTQ files. Quality of FASTQ files was checked by FastQC (version 0.11.7).^1^ Reads were mapped on the sugar beet reference genome KWS2320 (Dohm et al., 2013) with transcript annotation Beetset-2 (Minoche et al., 2015) using STAR version 2.2.1 (Dobin et al., 2013; Liao et al., 2014). FeatureCounts version 1.6.3 from Subread package was applied to count reads on transcript exons excluding annotated introns. Count data were normalized to TPM (transcripts per kilobase million), and expression data were further analyzed using the SARTools package (Varet et al., 2016) applying R software Version 3.4.3 (R Core Team, 2017), Bioconductor (Gentleman et al., 2004), and packages DESeq2 and EdgeR (Robinson et al., 2010; Love et al., 2014).

For analysis performed in this work, annotations of RNA-Seq results were assigned to the corresponding MapMan bincode found in the mapping file named “Ath_AFFY_STv1.1_TRANSCRIPT_CLUSTER_TAIR10_LOCUS.” Means were calculated over the log2FC over independent genotypes for each bincode and tissue, containing every log2FC≠0 without filtering for significant changes. Prior to calculation of log2FCs, CPM was filtered for values greater than zero. Violin plots used for analysis of RNA-Seq results were generated using BoxPlotR web page (Spitzer et al., 2014).

### Expression Analysis *via* RT-qPCR

Expression analysis of selected genes was performed by reverse transcription quantitative PCR (RT-qPCR) using the primers listed in Supplementary Table S2. RNA was extracted using the NucleoSpin(R) RNA Plant Kit (Machery-Nagel, Düren, Germany). RNA transcription into cDNA was performed using the qScript cDNA Synthesis Kit (Quantbio, Beverly, United States). Expression was analyzed in three biological replicates for each tested gene under each test condition. Relative expression was calculated *via* the ΔCT method relative to the expression of BvUGD1 (*BVRB_7g172340*; Supplementary Table S2). Detailed information on the primers used for quantification is listed in Supplementary Table S2.

### Metabolite Extraction and Quantification

Soluble metabolites were extracted from 20 to 50 mg freeze-dried plant material with 1ml 80% EtOH in sealed 1.5ml screw-lid reaction tubes at 80°C for 1h two times from four biological replicates per treatment. Extracts of the two extraction rounds were combined and vaporized in a vacufuge concentrator (Eppendorf, Hamburg, Germany). Evaporated pellets were resolved in _dd_H_2_O. Pellets remaining after extraction were washed with 80% EtOH and _dd_H_2_O for starch isolation. 200μl _dd_H_2_O was added to the pellet, and samples were autoclaved for 40min at 121°C. For hydrolytic cleavage, 200μl enzyme mix (5 U α-amylase; 5 U amyloglucosidase; 200mm sodium acetate; pH 4.8) was added to the pellet and incubated at 37°C for 4hours. Cleavage was terminated by heating to 95°C for 10min.

Sugars (glucose, fructose, and sucrose) and hydrolyzed starch concentrations were measured using a enzymatic assay based on the NAD +-dependent conversion of glucose-6-phosphate to 6-phosphoglukonolactone as described by (Stitt et al., 1989). Briefly, 20μl of extracted soluble sugars was mixed with 190μl of Premix [100mm HEPES (pH 5.7); 10mm MgCl_2_; 2mm ATP; 1mm NAD; 0.5 U Glucose-6-phosphate-dehydrogenase] and absorption at 340nm was measured in a Microplate Reader Infinite^®^ M Nano (Tecan, Männedorf, Switzerland). Hexokinase, phosphogluco-isomerase, and invertase were added sequentially for measurement, and absorbance was measured after addition of each enzyme until enzymatic saturation was reached. Sugar concentrations were then calculated based on the Lambert–Beer law for the NADH generated during enzymatic reaction.

Sugar alcohols, organic acids, raffinose, and galactinol were quantified *via* ion-chromatography as described by Ho et al., 2020. Briefly, a 761 Compact IC system (Metrohm, Herisau, Switzerland) was used. Organic acids were separated *via* the Metrosep organic acids 250/7.8 column, a Metrosep Organic Acids Guard/4.6 guard column (both Metrohm and Herisau, Switzerland), with 0,25 mm H_2_SO_4_ dissolved in ultrapure water used as eluent and 10mm LiCl as anti-ion. Sugar alcohols, raffinose, and galactinol quantification was done by ion chromatography on a Metrosep Carb 2–250/4.0 column using an 871 IC compact device (Metrohm and Herisau, Switzerland) followed by amperometric quantification. NaOH (0.1M) with sodium acetate (10mm) was used as the mobile phase. For peak analysis, the corresponding software Metrodata IC Net 2.3 SR5 by Metrohm (Herisau, Switzerland) was used.

Ions and amino acids were measured according to Duscha et al. (2020). Anions and cations were also measured in a 761 Compact IC system (Metrohm and Herisau, Switzerland). For anion concentration measurements, a Metrosep A Supp 4–250/4.0 column and a Metrosep A Supp 4/5 Guard/4.0 guard column (both Metrohm and Herisau, Switzerland) were used. 50mm H_2_SO_4_ was used as anti-ion, and 1,8 mm Na_2_CO_3_ together with 1,7 mm NaHCO_3_ dissolved in ultrapure water was used as eluent for anion measurement. For determination of cation concentrations, a Metrosep C4 150/4.0 column and a Metrosep C4 Guard/4.0 guard column (both Metrohm and Herisau, Switzerland) were used. The eluent consisted of 2mm HNO_3_ and 1,6 mm dipicolinic acid dissolved in ultrapure water. Amino acid concentrations were measured *via* high performance liquid chromatography in a Dionex (Dionex Softron, Germering, Germany) system, consisting of a Dionex ASI-100 automated sample injector, a Dionex P680 HPLC pump, and a Dionex RF2000 fluorescence detector. An AminoPac^®^ PA1 column (Dionex Softron, Germering, Germany) was used for separation of amino acids. 0.1M NaAc, 7mm Triethanolamine pH 5.2 was used as eluent. Samples were prepared for measurement by adding 60μl boric acid buffer (0.2M; pH 8.8) and 20μl 6-aminoquinolyl-N-hydroxysuccinimidyl carbamate (3mg dissolved in 1.5ml acetonitrile). Samples were vortexed and incubated at 55°C for 10min. Peaks were analyzed using the Chromeleon software (Thermo Fisher Scientific, Waltham, Massachusetts, United States).

### Histological Staining, Microscopy and Determination of Cell Size

Sections of sugar beet taproot tissue (0.5mm thickness) were made with a truffle slicer (Eppicotispai, Ornavasso, Italy) and were analyzed using a Leica MZ 10 F Binocular with a Plan APO 1.0x objective (Leica Microsystems, Wetzlar, Germany). Cell walls were stained using a Fuchsine-Chryosidine-Astra Blue (FCA) solution (Morphisto, Frankfurt am Main, Germany). Therefore, the sections were incubated in the staining solution for 5min and then washed with water, and staining was differentiated in 70% ethanol.

For tracking of phloem unloading, 5(6)-carboxyfluorescein diacetate (CFDA) was loaded onto source leaves as described by Mehdi et al. (2019). The cuticle of source leaves was scratched with sand paper. CFDA was prepared freshly from stock (stock: 10mgml^−1^ in acetone), by 1:7 dilution (v/v) with ddH_2_O and was pipetted on the roughened leaf surface. CFDA is non-fluorescent, but membrane permeable before the ester bonds is cleaved to yield the green fluorescent *CF* (carboxyfluorescein), which then is trapped within cells. 24h after loading, taproot sections were cut using a vibratome (125μm thickness), or by hand, cell walls were stained with propidium iodide and signals were detected upon excitation with 488nm with a TCS SP5II confocal microscope using a HCX PL APO lambda blue 20.0×0.70 IMM UV light objective (Leica, Weimar, Germany).

Mean sizes of pith and storage parenchyma cells (Supplementary Figure S7) were measured with ImageJ (Rasband, 1997–2018) using pictures of sugar beet cross sections. From each cross section, 10 cells were measured for each cell type. Overall, seven cross sections were analyzed, each cross section derived from an independent taproot representing one biological replicate.

### ROS Staining and Antioxidant Measurements

H_2_O_2_ and O_2_^−^ were detected in taproot sections according to (Fryer et al., 2002). Ascorbate concentrations were measured in three biological replicates according to Gillespie and Ainsworth (2007). Freshly harvested material was dissolved in 6% TCA and used for colorimetric detection of ascorbate at 525nm after the reaction with α-α-bipyridyl. Glutathione concentrations were measured in three biological replicates by the glutathione reductase-mediated reduction of DTNB (5,5-dithio-bis-(2-nitrobenzoic acid)) at 412nm according to Queval and Noctor (2007).

### Phylogeny

Phylogeny of GOLS and RS isoforms was calculated using the www.phylogeny.fr “one-click” mode (Dereeper et al., 2008). For graphical representation, phylogeny analysis from the Phylogeny.fr platform was loaded into FigTree v1.4.4.

### Statistical Analyses

PC analysis was performed using MetaboAnalyst 4.0 web server https://www.metaboanalyst.ca/ (Chong and Xia, 2018). Statistical analysis was performed using Student’s t-test, one-way, or two-way ANOVA. Student’s *t*-test was calculated using Microsoft Excel. One-way ANOVA with post-hoc Tukey HSD test was calculated using the calculator from Navendu Vasavada,^2^ while for two-way ANOVA, the two-way ANOVA with post-hoc Tukey HSD Test Calculator from Houssein Assaad^3^ (Assaad et al., 2015) was used.

## Results

### Sugar Beet Genotypes Respond Differently to Freezing Temperatures

Temperatures below freezing adversely affect sugar beet tissue. The resulting structural collapse of the affected cells leads to sucrose leakage from vacuoles and rotting of taproot tissue (Barbier et al., 1982). To learn about physiological and molecular responses that might mitigate harmful effects of freezing temperatures, we analyzed three genotypes (GT1, GT2, and GT3) with different freezing sensitivities from a panel of accessions that was tested for survival of freezing temperatures in field or greenhouse trials. In these tests, GT1 had low survival rates (SR; 14% in field and 11% in greenhouse trials), GT2 high SR (90% in field and 66% in greenhouse trials), and GT3 intermediate SR (77% in field and 56% in greenhouse trials). These rates showed a correlation of almost 100% between field and greenhouse trials and categorized GT1 as freezing sensitive, GT2 as freezing tolerant, and GT3 as moderately freezing tolerant (Figure 1A). For the experiments presented in this paper, we grew the three different genotypes under a fixed temperature profile in climate-controlled growth chambers and monitored their behavior in respect to the set temperature (Figure 1B). Freezing stress was applied until the temperature, measured 5cm below the surface of the potting soil, was decreased to 0°C (approximately 3days). After frost exposure, plants were transferred to 20°C for recovery. The severity of injuries in the three genotypes following treatment indeed mirrored their survival rates from the prior field and greenhouse trials. For instance, most of GT1 shoots collapsed during cold exposure and a comparably high number of GT1 plants did not recover and died after the freezing treatment (Figure 1C). Importantly, initial exposure to freezing temperature did not kill plants, but severely affected their performance during recovery at higher temperatures in a genotype-dependent manner (Figure 1C). Additionally, photosynthetic capacity was determined during cold and freezing treatments and a recovery phase at 12°C after freezing. We included the 12°C recovery treatment because it represents a milder and more naturally occurring temperature shift than a direct transition from 0°C to 20°C. Photosynthetic behavior during the recovery phase after freezing was analyzed after damage to plant tissue became apparent mainly after the transition to control growth conditions. Photosynthesis, measured as effective quantum yield of photosystem II [Y(II)], significantly declined during exposure to freezing temperatures in genotypes one and two, but strongest in freezing-sensitive GT1 plants (Figure 1D). In contrast, GT2 and GT3 plants were able to increase Y(II) to pre-freezing levels after retransfer to 12°C (Figure 1D). Consistently, GT1 plants showed higher non-photochemical quenching [Y(NPQ)] and unregulated energy dissipation [Y(NO)] in comparison with GT2 and GT3 plants during and after exposure to frost (Figure 1D). In addition, we recorded light curves for the different photosynthetic parameters at the corresponding temperatures (Supplementary Figure S1). With increasing light intensity, Y(II) of GT1 decreased faster than those of GT2 and GT3. Concomitantly, the Y(NPQ) percentages increased faster in of GT1 in comparison with GT2 and GT3 (Supplementary Figure S1). Following the freezing treatment, a large number of roots from GT1 plants displayed visible brownish rot in a defined region within the taproot neck, designated as pith (Figure 1E). With progression of the rotting, affected taproots lost structural integrity and plants died. Such changes, as observed above, can be evoked by high, damaging concentrations of ROS. To determine whether the low temperature exposure in general was associated with formation of ROS, we performed staining of taproot slices with diaminobenzidine (DAB) and nitroblue tetrazolium (NBT) to reveal accumulation of H_2_O_2_ and superoxide, respectively. Staining was most intense during or after freezing treatment, and DAB-derived dark brown or NBT-derived blue staining indicated that both types of ROS strongly accumulated in the vasculature and in the pith of taproots (Supplementary Figure S2). By staining, no clear differences in ROS emergence between the three genotypes could be revealed. Nevertheless, our observations showed that frost-induced tissue damage and emergence of ROS are clustered in the upper part of the sugar beet taproot, mainly in the pith tissue.

### The Sugar Beet Taproot Is Divided Into Two Distinct Tissue Zones With Different Sensitivities to Freezing

Almost a century ago, plant anatomist Ernst Artschwager noted the anatomic peculiarities of the pith tissue (Artschwager, 1926). Its anatomy differs from other internal taproot tissues, which mainly comprise the storage parenchyma (SP) and vascular tissue (Figure 2A). Our microscopic analysis confirmed Artschwager’s observation that the pith consists of spongiform parenchyma cells and revealed that pith cells are more than five times the size of parenchymatic cells alternating with xylem/phloem bundles in the distal parts of the beets (i.e., between the characteristic concentric rings that can be observed in transverse sections, mainly through the below-pith beet tissue; Figures 2B–D). While the storage parenchyma was streaked with vascular bundles, such long-distance transport tissue was absent from the pith, which was surrounded mostly by primary xylem (Figures 2C–E). The xylem in turn could be traced acropetally toward the vasculature of the oldest leaves and cotyledons, and basipetally to where some of the bundles converge in the central cylinder in below-pith root tissue (Figure 2F). Application of the phloem-mobile CFDA to source leaves and subsequent tracking of its green-fluorescent derivative *CF* in the taproot confirmed the occurrence of vascular bundles in storage parenchyma and, importantly, their absence from the pith region (Figures 2E,F).

To record differential responses in gene expression and metabolite accumulation in SP and pith, we manually separated both tissues. Because of the proximity of the pith to the shoot apical meristem (SAM; Figure 2A) and with a lack of known markers for the pith, we checked for accumulation of the mRNA of the SAM-marker *CLAVATA2* as indicator for successful enrichment of pith tissue (Figure 2G). To check whether SP and pith tissue are able to perceive cold and freezing treatment under our test conditions, expression of the low temperature response transcription factor *CBF3* was analyzed 2weeks after transfer to low temperatures (Figure 2H). Expression of *CBF3* did not differ between the three analyzed genotypes, but was significantly upregulated after cold and freezing treatment, especially in the pith tissue (Figure 2H). Indicating that both tissues are able to perceive cold and freezing treatment, and sensitivity of the pith tissue is not due to defects in primary cold sensing.

### ROS-Related Genes Are Upregulated in the Taproot Pith at Low Temperatures

**Figure 3:**
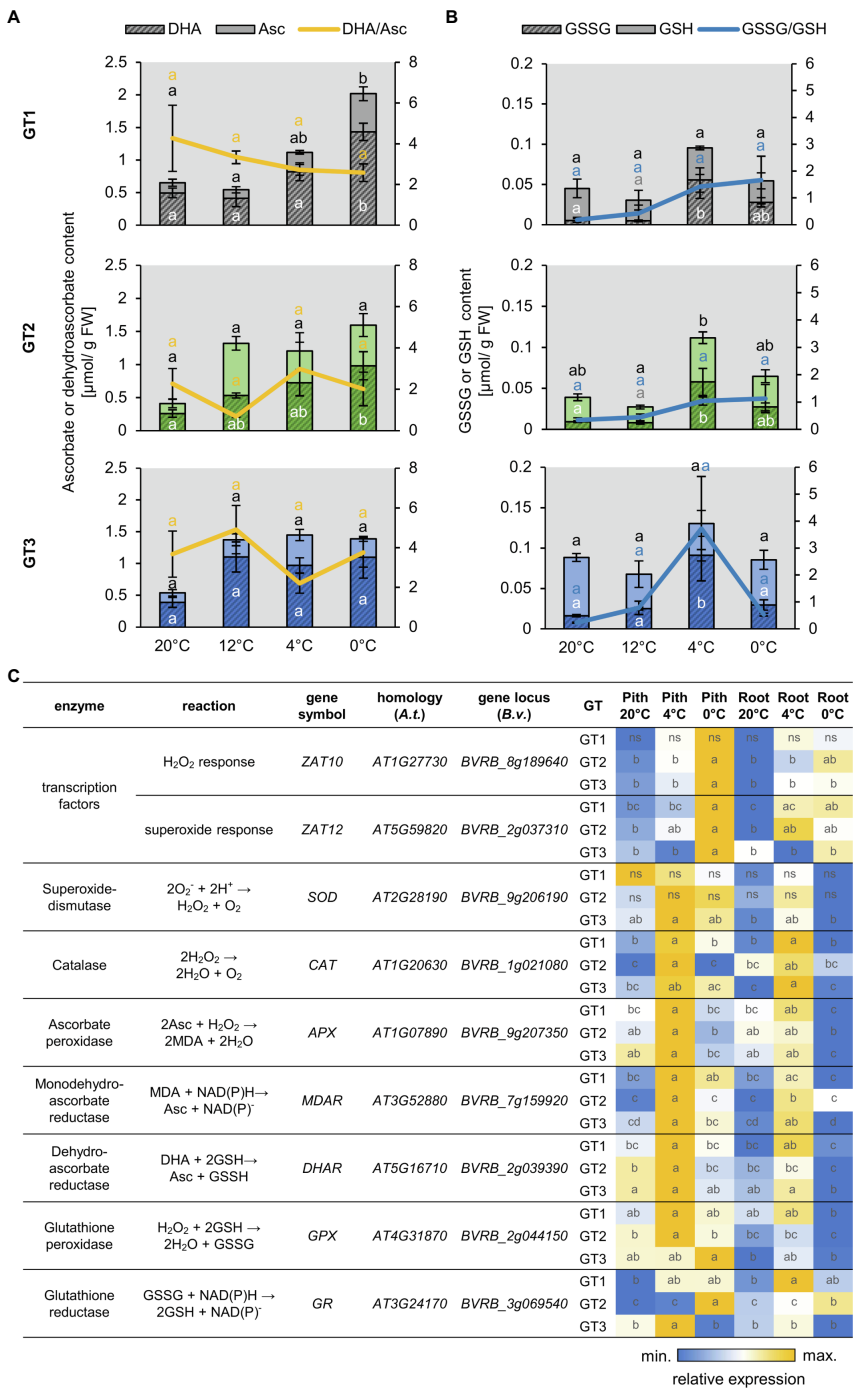
Reactive oxygen species (ROS)-related metabolite accumulation and gene expression. **(A,B)** Concentrations of ascorbate (Asc) and dehydro-ascorbate (DHA) **(A)** or reduced (GSH) and oxidized glutathione (GSSG) **(B)** in the pith of different sugar beet genotypes. Reduced form of the antioxidant depicted in light, oxidized form in dark color. Sugar beet plants were grown for 10weeks at 20°C and were successively transferred to 12°C, 4°C, and 0°C for 1week each. Three plants of each genotype were harvested at a given temperature and were analyzed for their antioxidant concentration. Error bars represent the standard error over the corresponding mean. Letters indicate the same level of significance calculated *via* one-way ANOVA with *post-hoc* Tukey HSD test with *p*<0.05. Black letters thereby represent significance level of reduced, white letters of oxidized compound. Yellow or blue letters indicate the significance level of the oxidized/reduced ratio correspondingly. **(C)** Heat map representation of RT-qPCR analysis of ROS relates genes in taproot pith and storage parenchyma (root) from the three genotypes grown at control (20°C) or low temperatures (4°C and 0°C). Values are compared over rows within each genotype, and data represent the mean of three independent biological replicates. Different letters within individual tiles denote significant differences between tested temperature conditions and tissues according to two-way ANOVA with post-hoc Tukey HSD testing (*p*<0.05).

The prominent staining of ROS in freezing-stressed taproots prompted us to analyze antioxidant levels and expression of ROS-related genes in pith and storage parenchyma of the different sugar beet genotypes (Figure 3). Low temperatures lead to a significant increase of the total ascorbate content but did not significantly alter the ratio of oxidized dehydro-ascorbate to reduced ascorbate in the pith tissues of GT1, GT2, and GT3 (Figure 3A). Glutathione also increased at low temperatures reaching highest levels at 4°C in the pith of all three genotypes. The ratio of oxidized GSSG to reduced GSH, serving as an indicator for the ROS stress level of the tissue, strongly increased with decreasing temperature, reaching its highest value at 0°C in GT1 and GT2, or at 4°C in GT3 (Figure 3B). Compared to the antioxidant contents in the pith, the contents in the storage parenchyma developed similarly with decreasing temperature, but the glutathione contents in the storage parenchyma were overall lower compared to the pith tissue of the different sugar beets (Supplementary Figure S3). Together with levels of antioxidants, gene expression was monitored in pith and SP during cold and freezing exposure (Figure 3C). Tissues were analyzed under control temperature (20°C), after 1week at 4°C, or after an additional 3days at 0°C. Most of the tested genes were upregulated in plants subjected to low temperature treatment, but the increase in expression was always greater in the pith compared to the SP. Genes encoding typical ROS-scavenging enzymes, like catalase, ascorbate peroxidase, and reductase, as well as glutathione peroxidase showed the highest expression at 4°C and were reduced at 0°C. Interestingly, expression of homologs to H_2_O_2_- and superoxide-responsive transcription factor genes *ZAT10* and *ZAT12* increased markedly at 0°C, but not at 4°C (Figure 3A) with highest upregulation observed in the pith tissue, indicating that ROS accumulation increased at 0°C.

In summary, these results indicate that low temperature-dependent ROS formation and response to ROS accumulation were most pronounced in the pith area of taproots and support the higher accumulation of ROS revealed by ROS staining (Supplementary Figure S2).

### The Pith Tissue Is Metabolically Separated From Taproot Parenchyma and Altered After Exposure to Subzero Temperatures

Besides the antioxidants ascorbate and glutathione, compounds such as sugars and amino acids contribute to ROS scavenging. Since ROS accumulation was pronounced in the pith region of taproots, we analyzed the low temperature-dependent accumulation of such metabolites and their spatial distribution between leaf, pith, and storage parenchyma tissues. In total, 36 compounds were measured and their contents are listed in Supplementary Table S1. A PC analysis, loaded with the 0°C/20°C fold-changes of these compounds, separated leaf, pith, and SP tissues (Supplementary Figure S4). The metabolic reaction of the storage parenchyma of the two more tolerant genotypes, GT2 and GT3, was similar, but differed from that of GT1 (Supplementary Figure S4). The separation of leaf tissue from pith and storage parenchyma was mainly based on changes in raffinose concentrations (Supplementary Figure S4). Different degrees of changes in starch content mainly contributed to the separation of pith and storage tissue (Supplementary Figure S4). Under control temperatures, the starch content of leaves exceeded that of pith and storage parenchyma about 10-fold (Supplementary Figure S5). After exposure to frost, starch decreased markedly in leaves (Supplementary Figure S5) consistent with the decline in photosynthetic activity (Figure 1). In contrast to leaves, where starch building blocks derive directly from photosynthesis, starch biosynthesis in taproots rather depends on import of glucose-6-phosphate into non-green plastids by glucose-6-phosphate translocators and consecutive conversion to glucose-1-phosphate by phosphoglucomutase (Turesson et al., 2014). The required glucose most likely derives from sucrose hydrolysis in the heterotrophic tissues. Freezing temperatures induced different changes in the starch content of pith and SP tissues. Pith starch contents decreased, and SP starch contents increased at 0°C non-significantly (Supplementary Figure S5), suggesting a differential activity of starch hydrolyzing and synthesizing enzymes in these two tissues. However, the contents of starch in pith and SP tissues were comparably low to the starch contents of leaves and almost neglectable to the high amount of stored sucrose in these tissues. It is therefore unlikely that starch contributes significantly to frost tolerance of sugar beet taproots (Turesson et al., 2014; Rodrigues et al., 2020). Freezing temperatures increased concentrations of glucose and fructose in leaves, but not significantly in pith and storage tissues (Supplementary Figure S5). Sucrose concentrations were highest in the SP and lowest in the leaves (Supplementary Figure S5). In both tissues, no significant changes in sucrose concentrations could be observed between control and freezing conditions, or between genotypes (Supplementary Figure S5). In the pith tissue, concentrations of sucrose differed at 20°C between genotypes, but fluctuated only marginally within and between genotypes at 0°C (Supplementary Figure S5).

### Genotype and Temperature-Dependent Accumulation of Inositol, Galactinol, and Raffinose

**Figure 4:**
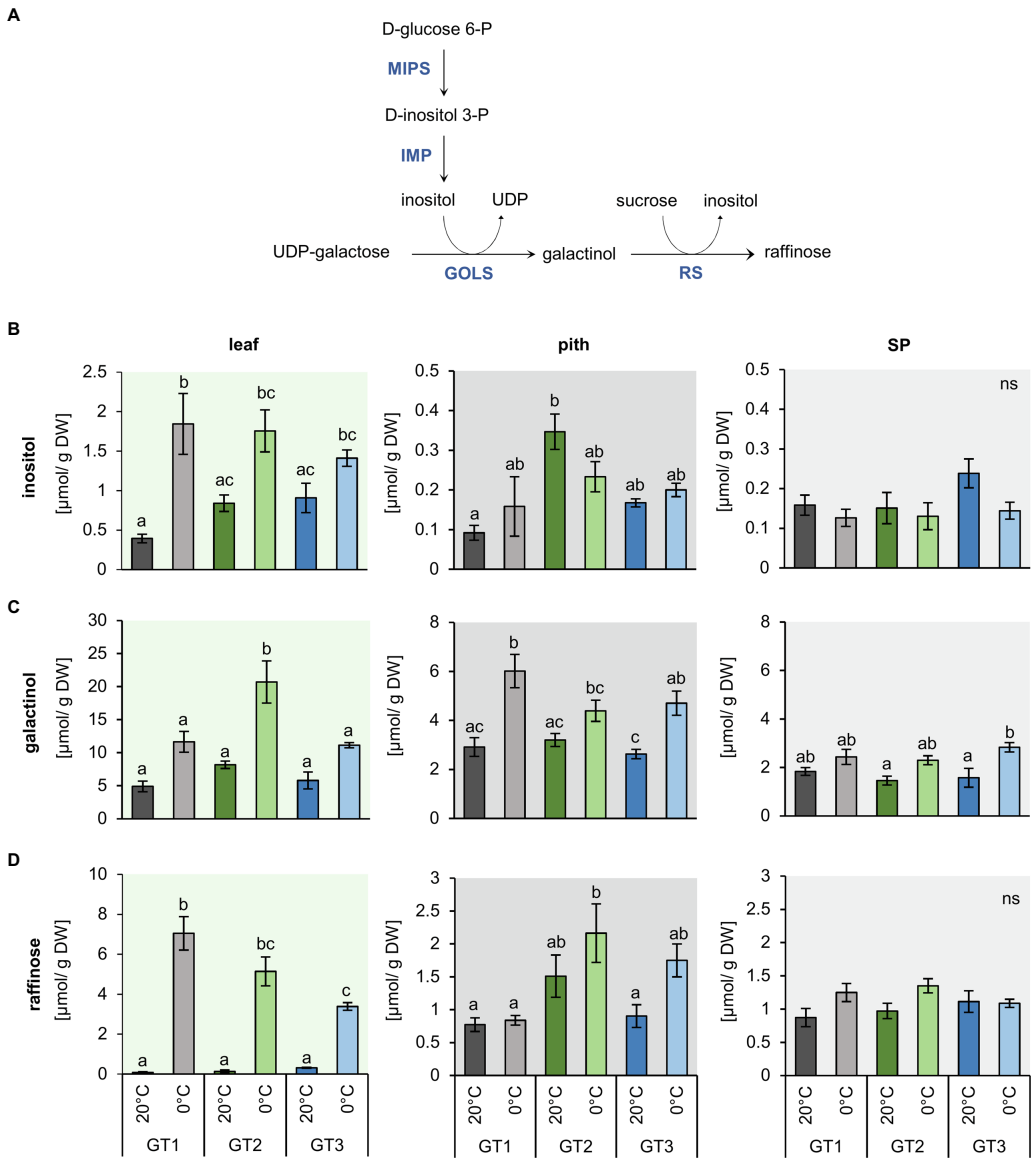
Inositol and raffinose synthesis and their concentrations in leaf, pith, and storage parenchyma tissue under control and freezing temperatures. **(A)** Schematic representation of inositol and raffinose synthesis in plants. **(B)** to **(D)** Raffinose and intermediates in raffinose synthesis were measured in plants grown at 20°C and plants transferred to 12°C, 4°C and harvested at 0°C soil temperature. Plants were dissected in the three different tissues leaf, pith, and root while harvesting at the corresponding temperatures. Inositol **(B)**, galactinol **(C),** and raffinose **(D)** concentrations represent the mean of four biological replications for each of the tested cultivars. Error bars represent the standard error over the corresponding mean. Letters indicate the same level of significance for each measured concentration, calculated *via* two-way ANOVA with *post-hoc* Tukey HSD testing with *p*<0.05.

As concentrations of glucose, fructose, and sucrose differed only marginally in pith tissues of the three genotypes after a shift from control to freezing conditions, we analyzed the contents of raffinose as well as of inositol and galactinol, which are precursors for raffinose synthesis (Figure 4A), because raffinose was shown to possess second-order rate constants for ROS scavenging, comparable or even higher than those of glucose, fructose, and sucrose and the antioxidant ascorbate (Nishizawa et al., 2008). The concentrations of inositol, galactinol, and raffinose were higher in the leaves compared to pith and SP (Figure 4). Inositol content of the leaves at 20°C was highest in GT2 and GT3. Upon 0°C exposure, inositol levels increased to comparable amounts in the leaf tissue of all three genotypes (Figure 4B). However, in the pith and SP, inositol levels remained unchanged upon exposure to freezing temperatures (Figure 4B). Like its precursor inositol (Figure 4A), concentrations of galactinol increased two- to three-fold in leaves of GT2 at 0°C (Figure 4C). Galactinol concentrations also increased in the pith and SP at 0°C, but only with significant changes in GT1 and GT3 for pith and GT3 for SP tissue. In the pith, galactinol accumulated at 0°C to concentrations twice as high as compared to the SP (Figure 4C). Notably, freezing temperatures boosted raffinose concentrations at least 10-fold, in GT3 leaves, and up to 80-fold, in GT1 leaves, in comparison with the corresponding leaf-tissue at 20°C (Figure 4D). In the pith tissue, raffinose content increased in GT2 from 1.5 to 2.3μmol/g DW and GT3 1.0 to 1.8μmol/g DW at 0°C. At 0°C, raffinose levels of GT2 exceeded those of GT1 two-fold in the pith. Interestingly, GT2, the cultivar with the highest freezing survival rates (Figure 1A), had twice as high raffinose concentrations in comparison with GT1, with the lowest freezing survival rates at 0°C (Figure 4D). Although non-significant, in SP, raffinose concentrations were slightly increased or remained unchanged at 0°C in comparison with 20°C (Figure 4D).

### Inositol, Galactinol, and Raffinose Biosynthesis Genes Are Differentially Expressed in Leaf and Taproot Tissue in Response to Freezing

**Figure 5:**
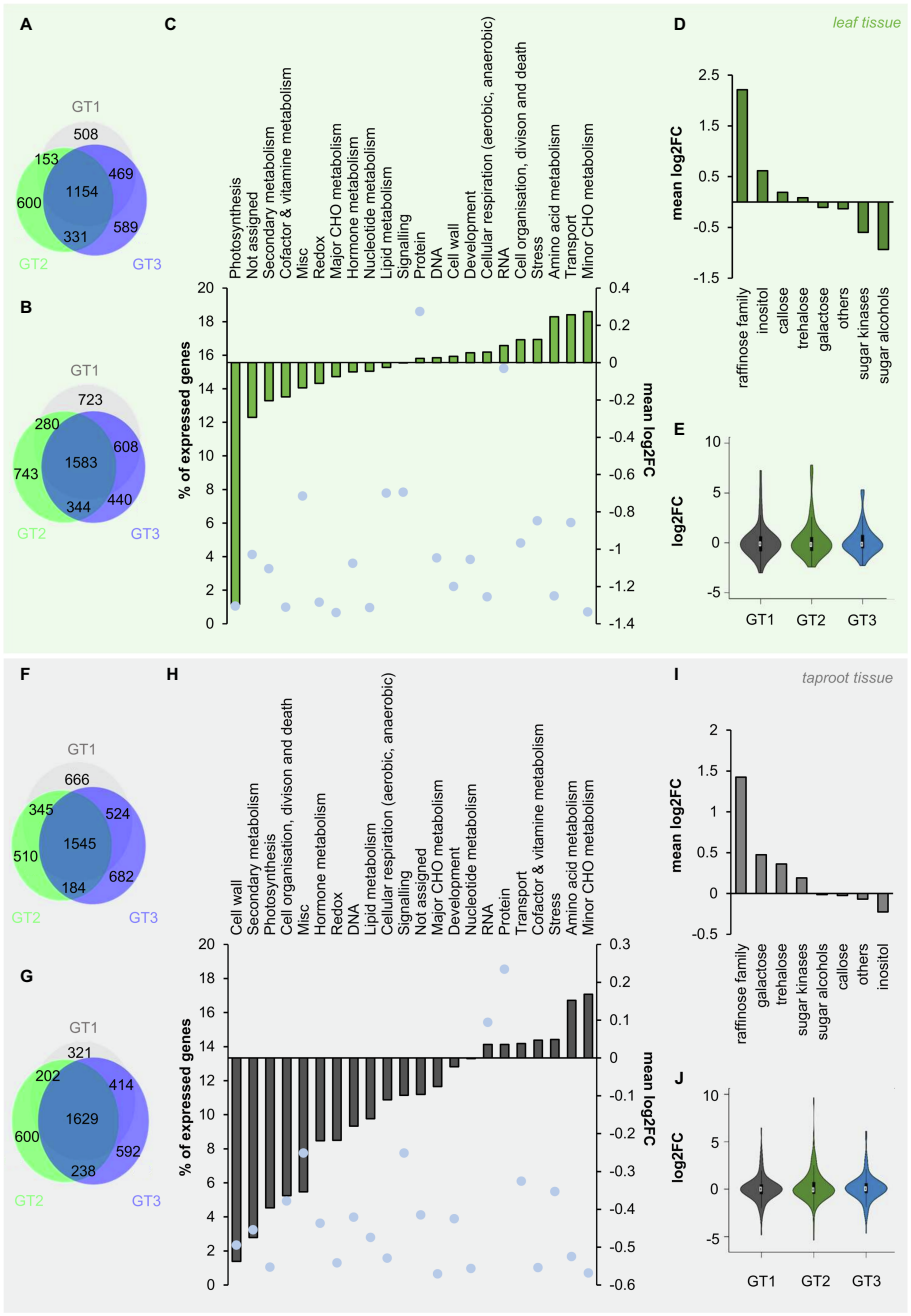
Changes in gene expression of *B. vulgaris* leaf and taproot tissues upon freezing treatment. (A, b; F, G) Venn diagrams of differentially expressed genes in leaves **(A,B)** and taproots (F, G). Numbers of up – (Log2FC≥1; A, F) or down – (Log2FC≤−1; B, G) regulated genes (with a FDR≤0.05) in leaves (A,B) or taproots **(F,G)** are given inside the circles of the diagram. Log2FC 0°C/20°C of all expressed genes with log2FC≠0 in leaf **(C)** and taproot tissue (H) were functionally grouped and plotted against the number of genes in each group given in percent of the total number of expressed genes (blue dots). **(D)** and (I) mean log_2_FC 0°C/20°C of genes with log2FC≠0 of different metabolic pathways included in the functional group of “minor CHO metabolism” in leaf (D) and taproot tissue **(I)**. Log2FCs represent the mean of the three different *B. vulgaris* genotypes GT1, GT2, and GT3, each measured in three biological replications for (C,D) in the leaf and **(H,I)** in the taproot. Log2FCs of genes included in the functional group of “minor CHO metabolism” are shown as violin plots for the corresponding tissues leaf **(E)** and taproot **(J)**. White circles show the medians, box limits indicate the 25th and 75th percentiles as determined by R software, whiskers extend 1.5 times the interquartile range from the 25th and 75th percentiles, and polygons represent density estimates of data and extend to extreme values in violin plots.

To reveal global patterns of gene expression in response to freezing stress, we performed comparative RNA-Seq analysis of leaf and taproot tissues of the three different genotypes at control and freezing temperatures. However, this analysis came with the limitation that we did not separate pith and storage parenchyma but instead analyzed total taproots. RNA-Seq reads were mapped to the sugar beet reference genome (Dohm et al., 2013) and revealed global rearrangement of gene expression in leaf and taproot tissue upon freezing exposure (Figures 5A,B,F,G).

To get a more general overview on regulation of different cellular pathways and reactions, RNA-Seq data were mapped into different functional groups, based on MapMan mapping. We then calculated the mean logarithmic fold change (log2FC) of all genes with log2FC≠0 mapped in each of these functional groups, without filtering for significant changes prior to analysis. This analysis revealed downregulation of genes involved in photosynthesis in leaf tissue at 0°C (Figure 5C). In contrast, minor CHO metabolism was the most prominently upregulated functional group in the leaf (Figure 5C) and also in the taproot tissue (Figure 5H). Minor CHO metabolism separates into the sub-bins raffinose, inositol, sugar alcohols, galactose, trehalose, callose synthesis, and sugar kinases. The mean log2FCs of genes involved in each of these sub-bins ranged from lowest values for sugar alcohols to highest for raffinose metabolism in leaves and from lowest mean log2FC for inositol to highest for raffinose metabolism in taproot tissue (Figures 5D–H). The latter opposite regulation of inositol and raffinose in taproots is interesting because inositol is essential for raffinose biosynthesis and the high log2FCs for raffinose in both leaves and taproots suggested increased utilization of carbon for raffinose biosynthesis under low temperatures. To reveal first genotype-dependent differences in the expression of minor CHO genes in the different genotypes, violin plots showed the distribution of log2FC over minor CHO genes in leaf and taproot tissues. While most of those genes show log2FCs between-2 and 2 in both leaf and taproot tissues upon freezing treatment (Figures 5E–J), in GT2, a few genes showed log2FC of up to 7 in the leaves and up to 10 in the taproot (Figures 5E–J). Overall, the number of highly upregulated genes in minor CHO metabolism of GT2 was higher than in GT1 and GT3, independent of the tissue (Figures 5E–J). To reveal possible genotype-specific regulation of genes involved in raffinose and its precursors synthesis, we extracted transcript levels for those genes upon exposure to 0°C (Table 1).

**Table 1:** Log2FC 0°C/20°C of expression of enzymes involved in inositol, galactinol, and raffinose synthesis of leaf and taproot tissue (root) of three differential *Beta vulgaris* genotypes GT1, GT2, and GT3.

Function	Gene identifier (RefBeet1.2)	Gene name	Annotation	Leaf GT1	Leaf GT2	Leaf GT3	Root GT1	Root GT2	Root GT3
Inositol biosynthesis	Bv6_135490_ioyj.t1	MIPS	Inositol-3-phosphate synthase *(M. crystallinum)*	3.5	**3.7**	3.2	1.0	**1.9**	0.5
	Bv3_059200_pzry.t1	IMP1	Type I inositol polyphosphate 5-phosphatase *(A. thaliana)*	−1.9	−1.0	−1.0	−3.3	−2.5	−3.0
	Bv4_097150_xtnp.t1	IMP2	Bifunctional phosphatase IMPL2, precursor *(A. thaliana)*	0.6	0.3	0.9	0.9	0.6	0.7
	Bv5_127150_wkgm.t1	IMP3	Phosphatase IMPL1, precursor *(A. thaliana)*	−1.2	−1.3	−1.1	0.0	0.0	0.2
	Bv8_184830_ufws.t1	IMP4	Inositol monophosphatase *(M. crystallinum)*	0.2	0.3	0.7	−0.6	−0.5	−0.6
	Bv9_217040_nmtf.t1	IMP5	Bifunctional phosphatase IMPL2, precursor *(A. thaliana)*	−0.3	−0.2	−0.4	−0.5	−0.6	−0.8
Galactinol biosynthesis	Bv5_122490_gtzu.t1	GOLS1	Galactinol synthase 1 *(A. thaliana)*	**7.2**	7.6	5.3	**2.3**	**1.8**	2.2
	Bv4_079980_rfmk.t1	GOLS2	Galactinol synthase 2 *(S. lycopersicum)*	5.7	**7.1**	5.0	0.6	**3.0**	1.1
	Bv4_080000_ugrr.t1	GOLS3	Galactinol synthase 2 *(S. lycopersicum)*	7.3	7.8	5.3	6.5	**10.0**	6.1
	Bv6_155630_dgxc.t1	GOLS4	Galacturonosyltransferase-like *(A. thaliana)*	−3.0	−2.4	−1.4	−4.8	**−5.4**	−4.6
Raffinose biosynthesis	Bv4_091140_xstd.t1	RS1	Galactinol-sucrose galactosyltransferase 1 *(A. thaliana)*	−2.9	−2.3	−2.3	−2.2	−1.6	−2.4
	Bv_006460_trdy.t1	RS2	Galactinol-sucrose galactosyltransferase 2 *(A. thaliana)*	0.9	0.8	0.9	2.1	**2.0**	1.8
	Bv7_177120_psfp.t1	RS3	Galactinol-sucrose galactosyltransferase 2 *(A. thaliana)*	0.6	0.5	0.3	−0.1	−0.3	−0.1
	Bv1_011340_dsoj.t1	RS5	Galactinol-sucrose galactosyltransferase *(P. sativum)*	**4.5**	**6.3**	4.8	3.3	**3.4**	**2.9**
	Bv8_193750_qhjs.t1	RS6	Galactinol-sucrose galactosyltransferase 6 *(A. thaliana)*	−0.2	−0.5	−0.2	0.3	0.5	−0.3

*Data have been extracted from the RNA-Seq analysis. Significant up- or downregulation at 0°C in comparison with the expression at 20°C is indicted by bold numbers. Significance was calculated using Student’s t-test with false discovery rate testing (p_adj_<0.1)*.

*De novo* inositol biosynthesis occurs *via* the enzymes myo-inositol phosphate synthase (MIPS) and inositol monophosphatase (IMP; Figure 4A; Loewus and Murthy, 2000). MIPS catalyzes the rate limiting step in inositol *de novo* synthesis (Donahue et al., 2010), isomerization of glucose-6-phosphate to inositol-3-phosphate. IMP then catalyzes de-phosphorylation of inositol-3-phosphate to inositol. In the *B. vulgaris* genome (Dohm et al., 2013), we identified one sequence with high homology to MIPS (*Bv*MIPS, Bv6_135490_ioyj.t1) and five sequences with high homology to IMP (*Bv*IMP1, Bv3_059200_pzry.t1; *Bv*IMP2, Bv4_097150_xtnp.t1; *Bv*IMP3, Bv5_127150_wkgm.t1; *Bv*IMP4, Bv8_184830_ufws.t1; *Bv*IMP5, Bv9_217040_nmtf.t1). Frost enhanced expression of *BvMIPS* in both, leaves and taproots, with the strongest induction occurring in the freezing-tolerant cultivar, GT2 (Table 1). Expression of the five *IMP* genes was differentially regulated upon 0°C, however, not in a genotype-dependent manner (Table 1).

Inositol can be fused to UDP-galactose to form alpha-D-galactosyl-(1->3)-1D-myo-inositol (galactinol) and UDP. This reaction is catalyzed by GOLS and is required for subsequent biosynthesis of raffinose (Figure 4A). We identified four genes with homology to GOLS from tomato (*Solanum lycopersicum*) or *Arabidopsis* in the sugar beet genome (Table 1; Supplementary Figure S6). The expression of three of the four *GOLSs* (*BvGOLS1 to 3*) was strongly upregulated at 0°C in leaves and in taproots. The log2FCs for *GOLS2* and *GOLS3* in leaves and especially in the taproot of GT2 exceeded those of the less freezing-tolerant genotypes GT1 and GT3 (Table 1). The gene coding for a fourth GOLS isoform, *GOLS4*, was downregulated at 0°C, independent of the tissue and genotype (Table 1). Phylogenetic analysis revealed that *BvGOLS4* (Bv6_155630_dgxc.t1) was only distantly related to the other sugar beet GOLS isoforms (Supplementary Figure S6) and therefore may not encode a GOLS relevant for the plants freezing response.

The final step in raffinose biosynthesis is the transfer of galactose from galactinol to sucrose by the enzyme raffinose synthase (RS). During this reaction, inositol is released from galactinol (Figure 4A). We identified seven RS isoforms in the sugar beet genome (Supplementary Figure S6). Two isoforms, *BvRS4* and *BvRS7*, were not changed in their expression. Of the remaining 5 isoforms, two RS, *BvRS2* and *BvRS5*, were upregulated in the leaf and taproot tissue at 0°C (Table 1). Overall, *BvRS5* (*Bv1_011340_dsoj.t1*), the closest homolog to *AtRS5* (Supplementary Figure S6), showed the highest upregulation at 0°C (Table 1). Similar to the GOLS genes, upregulation was strongest in freezing-tolerant GT2 plants, independent of the tissue (Table 1).

### Raffinose Biosynthesis Genes Are Induced in Pith Tissues at Low Temperatures in a Genotype-Dependent Manner

**Figure 6:**
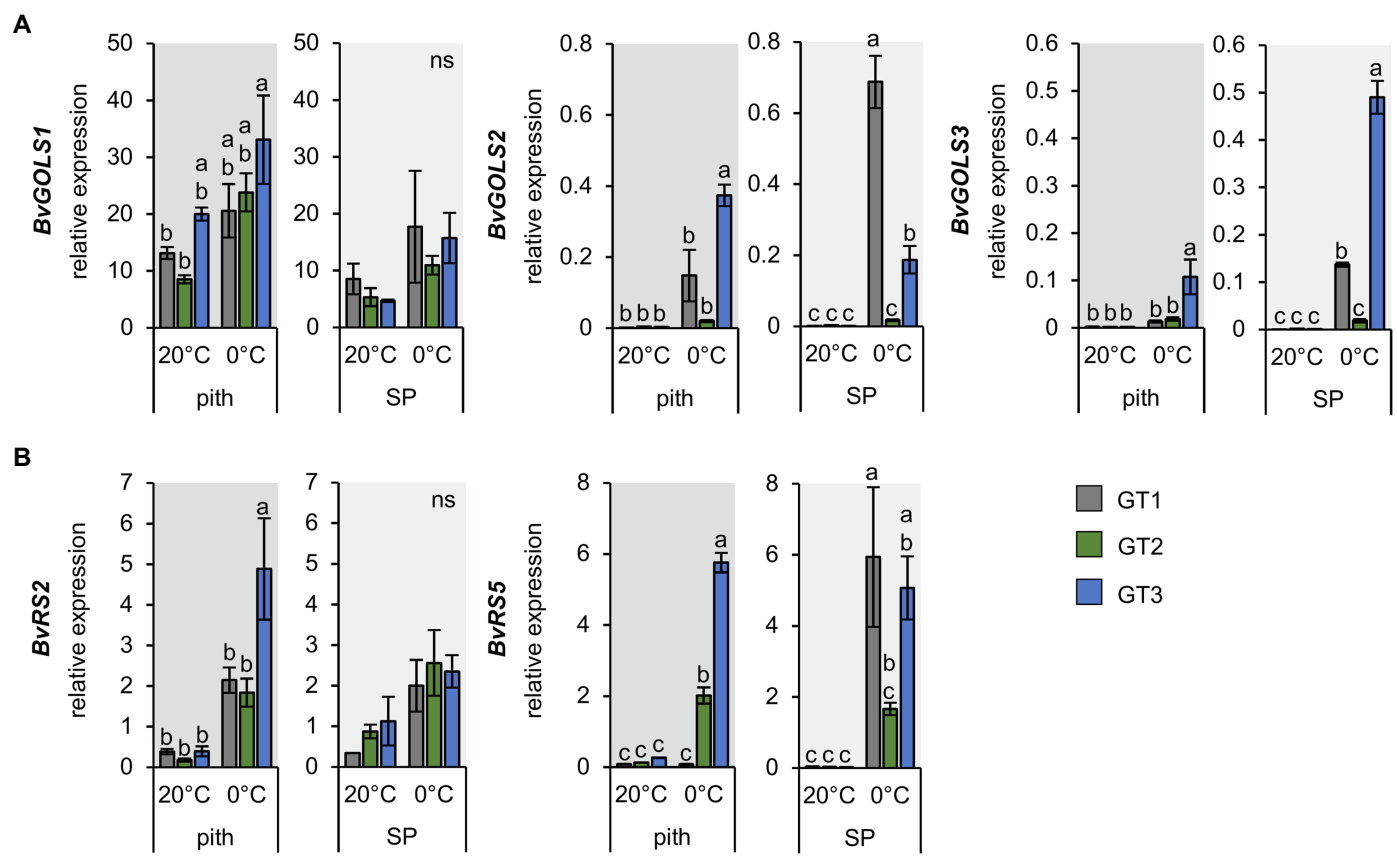
Expression of *GOLS* and *RS* isoforms in pith and storage parenchyma samples of three contrasting *B. vulgaris* cultivars. Relative expression of *GOLS* **(A)** and *RS* isoforms **(B)** in the pith and storage parenchyma at 20°C and freezing treatment at 0°C. Bars represent means from three biological replicates ± standard error. Different letters indicate significant differences between genotypes within a tissue according to two-way ANOVA with *post-hoc* Tukey HSD testing (*p*<0.05).

The RNA-Seq analysis suggested that raffinose metabolism is important in leaf and taproot tissues during the freezing response of sugar beet plants (Figure 5 and Table 1). Because of the different sensitivities of the pith of the three genotypes to freezing, we analyzed the expression of galactinol and raffinose biosynthesis-related genes in taproot tissues with RT-qPCR (Figure 6). Among GOLS, *GOLS1* was the most abundant isoform in the pith and storage parenchyma (Figure 6A); however, upregulation of gene expression in terms of fold-change was strongest for *GOLS2* and *GOLS3* at 0°C (Figure 6A). Expression of *GOLS2* and *GOLS3* was highest in GT1 and GT3 at 0°C (Figure 6A). The two RS isoforms *RS2* and *RS5* showed comparable abundance in the pith of all three genotypes at control temperatures. Abundance of *RS2* was slightly higher in the SP, while *RS5* abundancy in the SP was comparable to the pith at 20°C (Figure 6B). Additionally, the upregulation of RS expressions in terms of fold-change in the pith was comparable between *RS2* and *RS5* expression in GT2 and GT3. Overall, GT3 showed the highest fold-change upregulation of *RS2* and *RS5* in the pith (Figure 6B). In GT1 pith tissue, upregulation of *RS2* expression was comparable to that in GT2 (Figure 6B). While *RS2* was upregulated in the pith of GT1 upon exposure to 0°C, GT1 pith tissue lacked the ability of upregulation of *RS5* upon exposure to freezing temperatures (Figure 6B). While *RS5* expression increased more than 15- and 20-fold in the pith of GT2 and GT3 respectively, expression remained unaltered in the pith of the less tolerant GT1 upon 0°C (Figure 6B). GT1 failed to induce *RS5* in the pith tissue in comparison with GT2 and GT3, and induction of *RS5* expression was highest in SP of this genotype (Figure 6B). Taken together, expression of *RS* genes in the pith was upregulated to a greater extent in the freezing-tolerant cultivars GT2 and GT3, and to a lower extent in sensitive GT1 (Figure 6B), which is in line with the low raffinose concentrations in the pith of this genotype at 0°C (Figure 4D).

## Discussion

**Figure 7:**
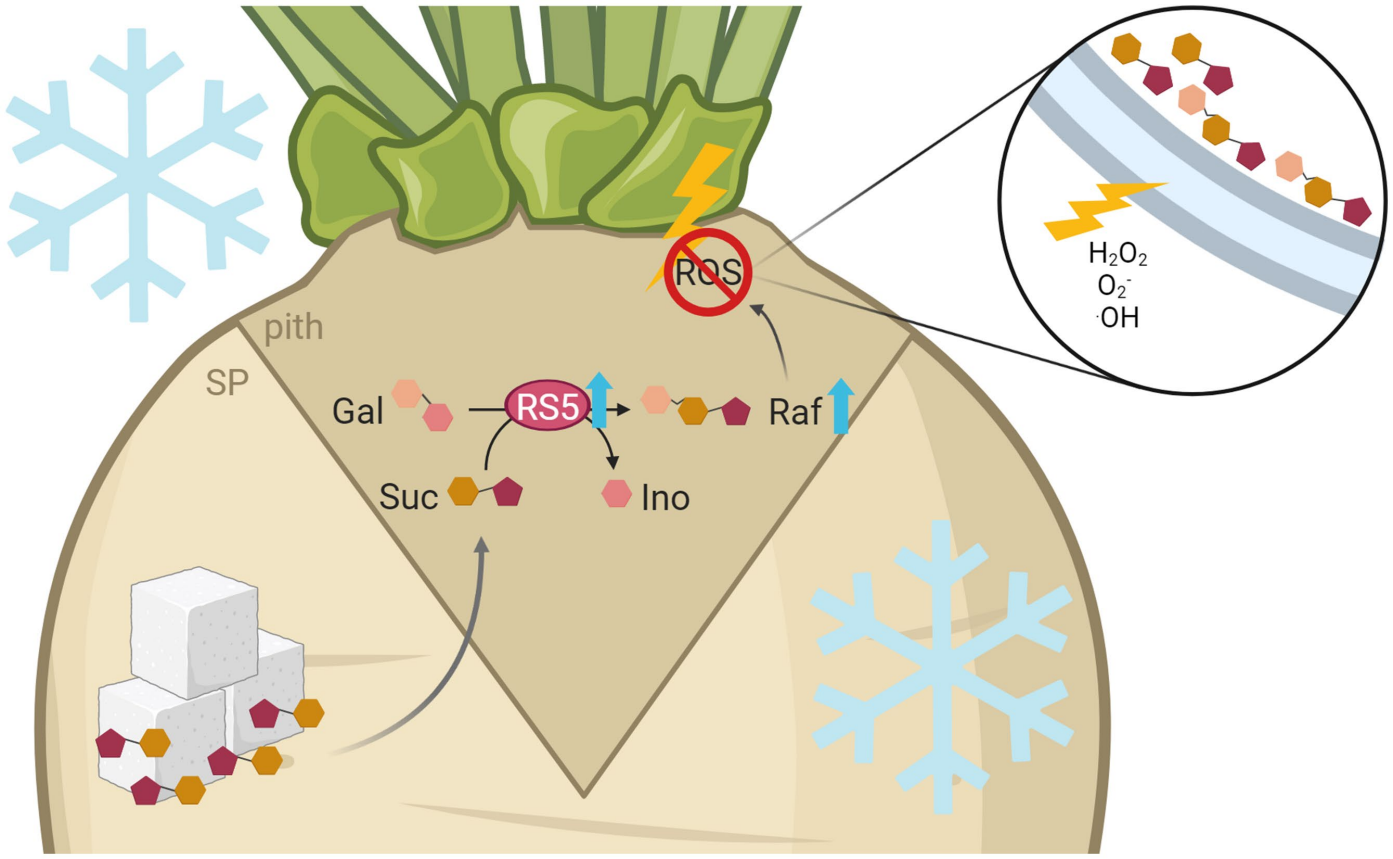
Model of raffinose-mediated freezing tolerance in the sugar beet pith tissue. Freezing temperatures lead to increased expression of raffinose synthesizing enzymes, especially *BvRS5*, in more tolerant cultivars. This results in higher raffinose contents in the pith. Frost exposition leads to high, damaging concentrations of H_2_O_2_, O_2_^−^, and ·OH especially in the pith tissue of sugar beet. There, high concentrations of ROS lead to damage of membranes by lipid peroxidation, which ultimately results in a structural collapse and therefore a brownish rot. Raffinose can protect the frost damage prone pith tissue against those harmful effects by a direct reaction with ROS. *This figure was created with* *BioRender.com*.

Low temperatures above freezing cause drastic changes in sugar beet physiology and development, leading to a change in organ identity from sink to source in taproots or *vice versa* in leaves (Rodrigues et al., 2020). In contrast to such developmental reprogramming, freezing temperatures can rather have damaging effects on internal tissues of sugar beet taproots (Figures 1C,E). In-depth analyses of the affected tissue and the molecular processes involved that cause or mitigate such damage are of great interest to breeders seeking to improve sugar beet winter survival. We summarize the findings discussed in this section in the model presented in Figure 7.

Transcriptomic analysis of sugar beet leaves and taproot revealed that genes of the minor CHO metabolism were strongly upregulated in both tissues (Figure 5 and Table 1), while genes involved in photosynthesis were massively downregulated in leaves by freezing treatment (Figure 5). The latter observation together with the decline in photosynthetic activity (Figure 1D) is in line with our recent results, where low above-zero temperatures evoked similar responses (Rodrigues et al., 2020). Such effects, which have also been observed at the protein level in leaves of cold-stressed *Arabidopsis* plants (Fanucchi et al., 2012; Janmohammadi et al., 2015), could be a direct effect of electron overflow at photosynthetic reaction centers caused by a slowdown of enzymatic Calvin-Benson cycle reactions in the cold and can be interpreted as an adaptive mechanism to avoid damage to photosynthetic components. Excess electrons from PSI can in fact cause direct oxidation of oxygen and lead to the generation of ROS in the chloroplast and subsequent damaging of cellular components upon cold treatment (Strand et al., 1997; Gechev et al., 2006; Choudhury et al., 2017; Pommerrenig et al., 2018).

In contrast to leaves, ROS in heterotrophic tissue does not predominantly originate from chloroplasts, but from reactions in the mitochondria, cytosol, or cell wall (Mittler et al., 2004). In our experiments, low temperatures induced the formation of ROS in the heterotrophic taproot tissue of sugar beet (Figure 1). That ROS might have an acclimating effect under cold conditions, as ROS detoxifying enzymes, like catalase or superoxide dismutase, were highly upregulated upon 4°C (Figure 3; Hossain et al., 2015). However, decrease in expression of those enzymes and simultaneous increase of the H_2_O_2_ and O2-marker genes *ZAT10* and *ZAT12* upon freezing temperatures indicate that upon 0°C, ROS rather accumulates to damaging concentrations, than having priming functions. The high ROS accumulation in the pith, as indicated by DAB and NBT staining (Supplementary Figure S2) and strong upregulation of ROS marker genes *ZAT10* and *ZAT12* (Figure 3), therefore, could be the cause of the observed tissue damage.

The accumulation of hydroxyl group-rich molecules, such as soluble sugars, sugar alcohols, or raffinose, can diminish those harmful effects of ROS. This is because these compounds have second-order rate constants for detoxification of hydroxyl radicals that are higher than those of common antioxidants, such as ascorbate or glutathione (Nishizawa-Yokoi et al., 2008). Moreover, high concentrations of these metabolites have been shown to stabilize ascorbate and glutathione concentrations, which are involved in detoxification of ROS (Nishizawa-Yokoi et al., 2008; Keller et al., 2021).

We found that concentrations of the monosaccharides glucose and fructose increased in the leaf tissue at 0°C (Supplementary Figure S5), in a manner similar to other plant species in which these sugars accumulate during cold treatment (Usadel et al., 2008; Klemens et al., 2014; Ho et al., 2020). The concentrations of glucose and fructose were highest in the leaves, whereas concentrations of sucrose were highest in the heterotrophic storage parenchyma (Supplementary Figure S5). The freezing-sensitive pith tissue contained comparably lower concentrations of these sugars. Our CFDA staining experiments demonstrated the organization of the phloem in vascular rings surrounding the pith (Figure 2) and explained why sugars translocated *via* and unloaded from the phloem cannot directly enter the pith tissue (Figure 2; Artschwager, 1926; Jahnke et al., 2009; Metzner et al., 2014). The sugar concentrations did not significantly differ between the pith tissues of the sugar beet genotypes with different sensitivity to freezing, suggesting that factors other than the above sugars contributed to freezing tolerance.

As outlined above, we observed that galactinol and raffinose synthesis was highly upregulated upon freezing temperatures (Figure 5 and Table 1). Raffinose accumulates during cold stress (Taji et al., 2002; Saito and Yoshida, 2011), and genes encoding corresponding biosynthesis enzymes have been reported to be upregulated during cold treatment in many different plant species (Sprenger and Keller, 2000; Cunningham et al., 2003; Zuther et al., 2004; Egert et al., 2013; Zhuo et al., 2013). In addition to these studies, our analysis revealed tissue-specific differences of the expression of raffinose-related genes and the accumulation of the corresponding metabolites in sugar beet.

Among the four genes coding for GOLS (Table 1 and Supplementary Figure S6), three were upregulated at freezing temperatures, including *GOLS2* and *GOLS3* (Table 1 and Supplementary Figure S6), confirming results from *Arabidopsis*, where *GOLS2* and *GOLS3* were upregulated by both drought and cold stress (Taji et al., 2002; Maruyama et al., 2009; Shen et al., 2020). Two genes encoding raffinose synthases, *BvRS2* and *BvRS5*, were also upregulated at freezing temperatures (Table 1). In *Arabidopsis*, RS2 was shown to possess α-galactosidase activity (Peters et al., 2010), while RS5 was proven to be a raffinose synthase that is upregulated in the cold (Egert et al., 2013). Interestingly, *BvRS5* was highly upregulated in the pith tissue of the two freezing-tolerant cultivars, GT2 and GT3, but not in freezing-sensitive GT1 (Figure 6B). Consistently, raffinose level in the pith of the freezing-tolerant genotypes differed from those of the sensitive GT1 genotype (Figure 4D). In GT2, highest upregulation of *GOLS* and *RS* genes (Table 1) and strongest accumulation of raffinose in the pith (Figure 4) correlated with the highest freezing tolerance among the three genotypes (Figure 1A). GT3 plants exhibited the strongest freeze-dependent increase in raffinose in the pith coinciding with strong upregulation of *RS5* specifically in the pith tissue (Figure 6B and Figure 4D). In GT1, on the other hand, raffinose concentrations and expression of raffinose synthase in the pith were not significantly elevated by freezing stress (Figure 4D and Figure 6B). These data are consistent with previous reports, indicating that raffinose levels are increased at cold and freezing temperatures and suggest that pith survival may be related to high raffinose concentrations.

Although raffinose is synthesized in the cytosol, about 20% of cellular raffinose is located to the chloroplasts and its concentration is increased even further at low temperatures (Schneider and Keller, 2009; Knaupp et al., 2011). However, most of the cellular raffinose is located in the vacuole and reaches about 60% of cellular raffinose in cold-acclimated *Arabidopsis* plants (Knaupp et al., 2011). Compared to leaves, taproot tissue does not contain chloroplasts, and the vacuole is probably the main site for raffinose accumulation. There, raffinose might accumulate near the tonoplast together with other carbohydrates. Excess cytosolic H_2_O_2_ that passes through the tonoplast, or H_2_O_2_ originating from superoxide produced by tonoplast-localized NADPH oxidases, could then react with raffinose, similar to the reaction proposed for fructans (Peshev et al., 2013; Matros et al., 2015), to protect the tonoplast from ROS-mediated lipid peroxidation.

In addition to its important role in ROS detoxification, raffinose can assist in the osmotic adjustment and thereby maintain cell turgor during water loss due to freezing (ElSayed et al., 2014). The higher raffinose concentrations of GT2 and GT3 in comparison with freezing-sensitive GT1, especially in the pith tissue, suggest a beneficial contribution of raffinose for freeze protection of taproots. Especially, juvenile roots before the beginning of sucrose storage phase are very susceptible to frost. In the future, it will be tempting to test the hypothesis of whether high accumulation of raffinose can protect young sugar beet plants against harmful ROS production and freezing damage. Such experiments would require the generation of transgenic plants and the usage of pith-specific promotors that are active at young developmental stages. Their identification should therefore be an objective for future biotechnological strategies aimed at increasing RFO synthesis in this sensitive tissue. Latter strategy will also help to circumvent a trade-off between RFO synthesis and sucrose accumulation in the storage parenchyma.

In *Arabidopsis*, ICE-CBF/DREB1 regulation is the main low temperature-responsive pathway leading to raffinose accumulation (Janmohammadi et al., 2015), and in our experiments, sugar beet *CBF3* was highly expressed at freezing temperature in the pith (Figure 2H). It is tempting to speculate that the regulation of the same pathway might cause an upregulation of specific GOLS and RS isoforms also in *B. vulgaris*, as the closest sugar beet homologs to *AtGOLS2*, *AtGOLS3*, and *AtRS5* all showed similarly high upregulation upon freezing temperatures (Table 1; Supplementary Figure S6). Our findings and datasets presented in this paper might help future studies in identifying specific transcription factors of galactinol and raffinose biosynthesis in sugar beet.

## Data Availability Statement

The datasets presented in this study can be found in online repositories. The names of the repository/repositories and accession number(s) can be found in the article/Supplementary Material.

## Author Contributions

FL, WK, KH, US, HN, and BP designed the research. IK, CM, CR, DK, WZ, OC, KF-W, and BP performed the research. IK and BP analyzed the data and wrote the paper. All authors contributed to the article and approved the submitted version.

## Conflict of Interest

OC, KF-W, WK, and FL are employed by KWS SAAT SE & Co. KGaA. This study received funding from KWS SAAT SE & Co. KGaA. The funder had the following involvement with the study: selection and propagation of germplasm material and plant growth for survival rate testing. All authors declare no other competing interests.

## Publisher’s Note

All claims expressed in this article are solely those of the authors and do not necessarily represent those of their affiliated organizations, or those of the publisher, the editors and the reviewers. Any product that may be evaluated in this article, or claim that may be made by its manufacturer, is not guaranteed or endorsed by the publisher.

## Footnotes

^1^ www.bioinformatics.babraham.ac.uk

^2^ https://astatsa.com/OneWay_Anova_with_TukeyHSD

^3^ https://houssein-assaad.shinyapps.io/TwoWayANOVA/
