# Supplementary material for: Cold-Triggered Induction of ROS- and Raffinose Metabolism in Freezing-Sensitive Taproot Tissue of Sugar Beet
Source: Front Plant Sci. 2021 Sep 3;12:715767. doi: 10.3389/fpls.2021.715767 (PMC8446674; doi:10.3389/fpls.2021.715767)
Supplement: Supplementary file 1 [file Data_Sheet_1.DOCX]

Supplementary Material

**
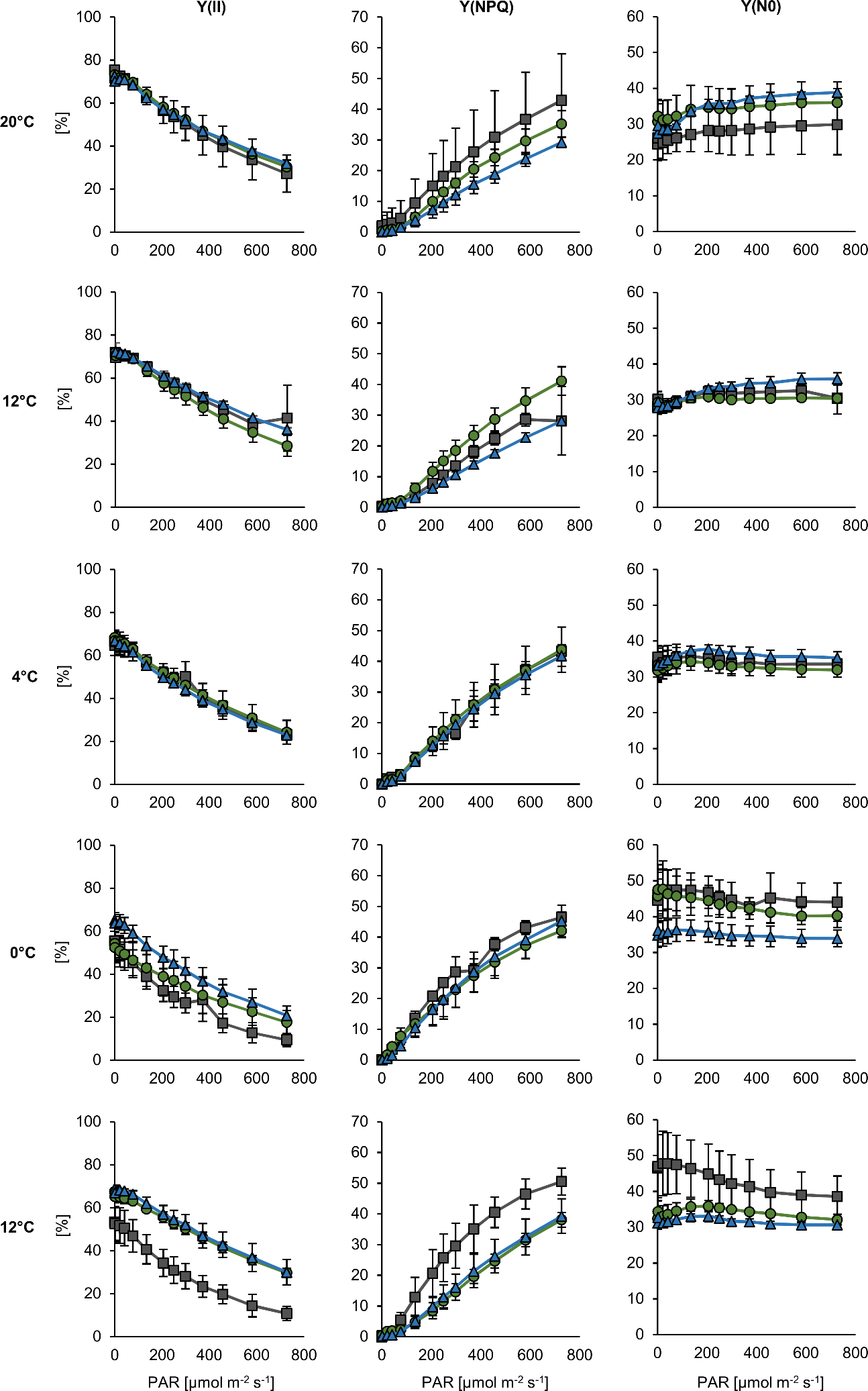
**

**Supplemental Figure S1: Light curves for the photosynthetic parameters Y(II), Y(NPQ) and Y(NO).** Light curves were measured by incrementally increasing light pulses with an intensity from PAR 0 (μmol photons m-2 s-1) to PAR 726 on leaves of the three different genotypes at 20°C, after 7 days at 12°C, 7 days at 4°C, 7 days at 0°C and after recovery at 12°C for 7 days. Quantum yield of photosynthesis [Y(II)] and non-photochemical quenching [Y(NPQ)] and non-regulated energy dissipation [Y(NO)] were calculated. Values represent the mean of three biological replicates. Error bars represent the standard error of the corresponding mean.

**
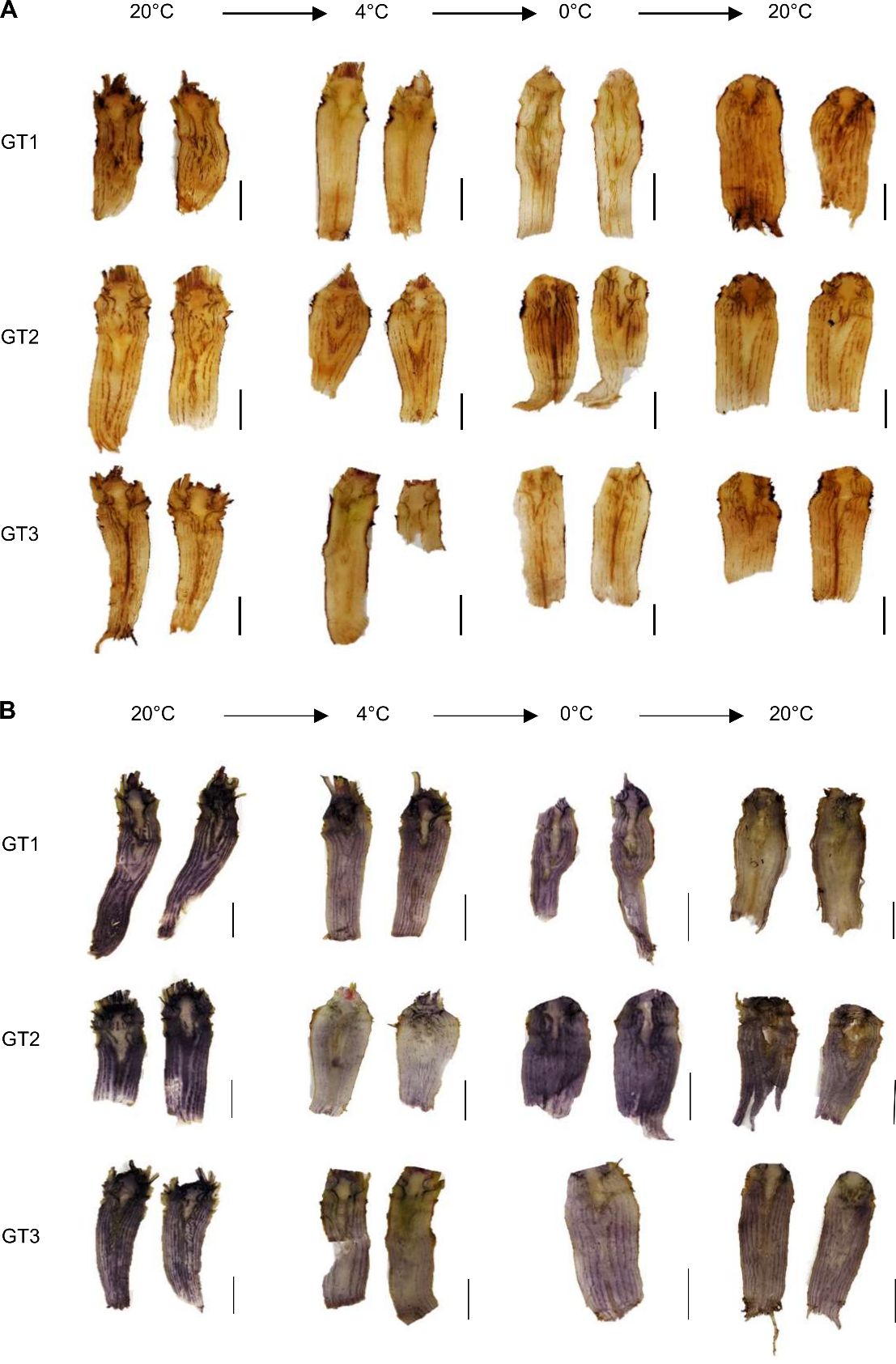
**

**Supplemental Figure S2: DAB and NBT staining of sugar beet taproot sections.** (A) DAB staining is indicative for H_2_O_2_ accumulation. (B) NBT staining is indicative for superoxide accumulation. 10-week old taproots were sectioned and stained after growth under control conditions or at 4°C, 0°C and recovery after freezing at 20°C. Scale bars represent 1cm.

**
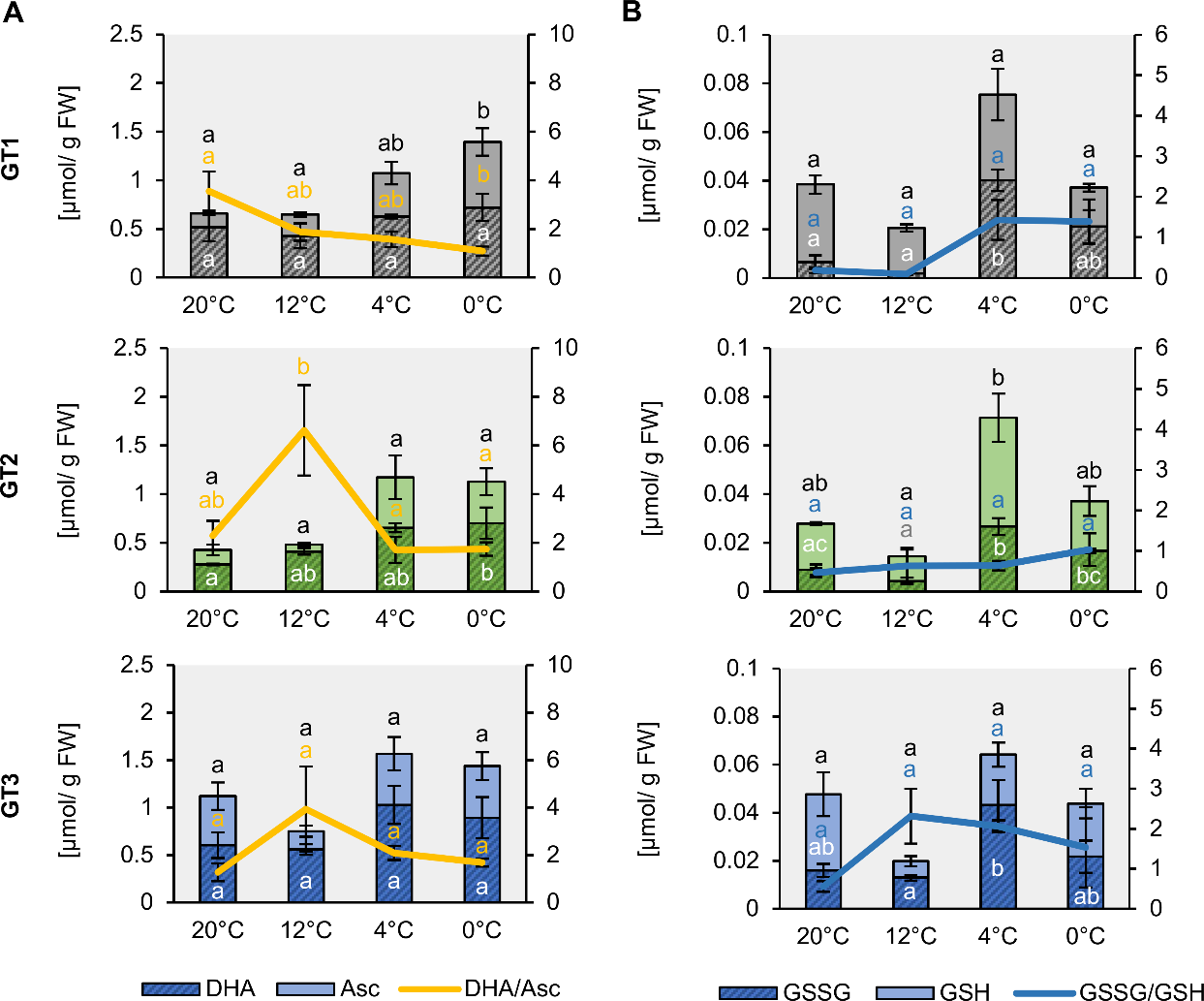
**

**Supplemental Figure S3: Antioxidant level of storage parenchyma.** (A) and (B) concentrations of ascorbate (Asc) and dehydro-ascorbate (DHA) (A) or reduced (GSH) and oxidized glutathione (GSSG) (B) in the storage parenchyma of different sugar beet genotypes. Reduced form of the antioxidant depicted in light, oxidized form in dark color. Error bars represent the standard error over the corresponding mean. Letters indicate the same level of significance calculated via one-way ANOVA with post hoc Tukey HSD test with p< 0.05. Black letters thereby represent significance level of reduced, white letters of oxidized compound. Yellow or blue letters indicate the significance level of the oxidized/reduced ratio correspondingly.

**Supplemental Table S1.** Metabolite concentrations of sugar beet leaf, pith and storage parenchyma (root) tissues. Mean and standard error of measured metabolites in every tissue and temperature analyzed, given in µmol/g DW. Means were calculated over four biological replications.


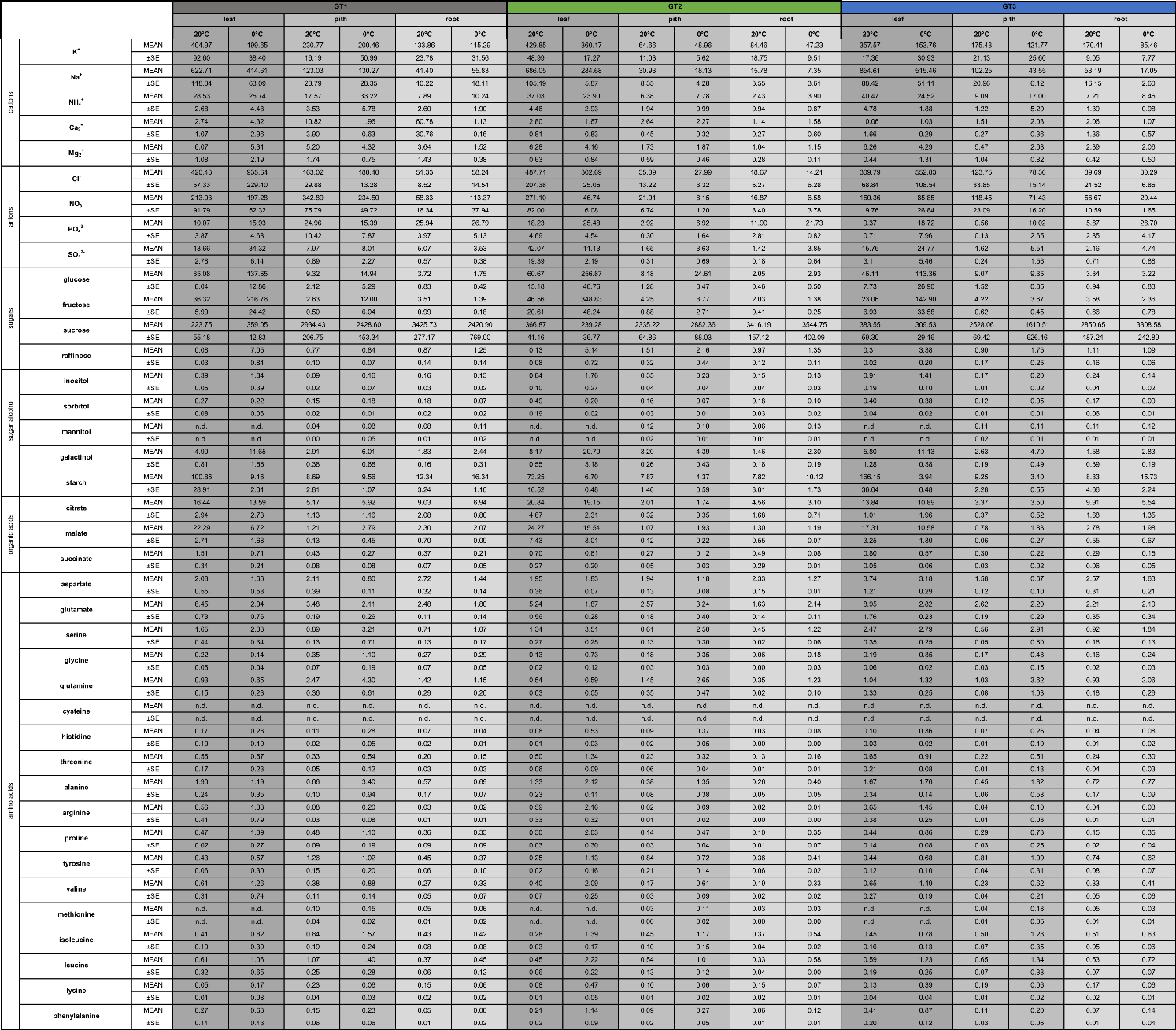


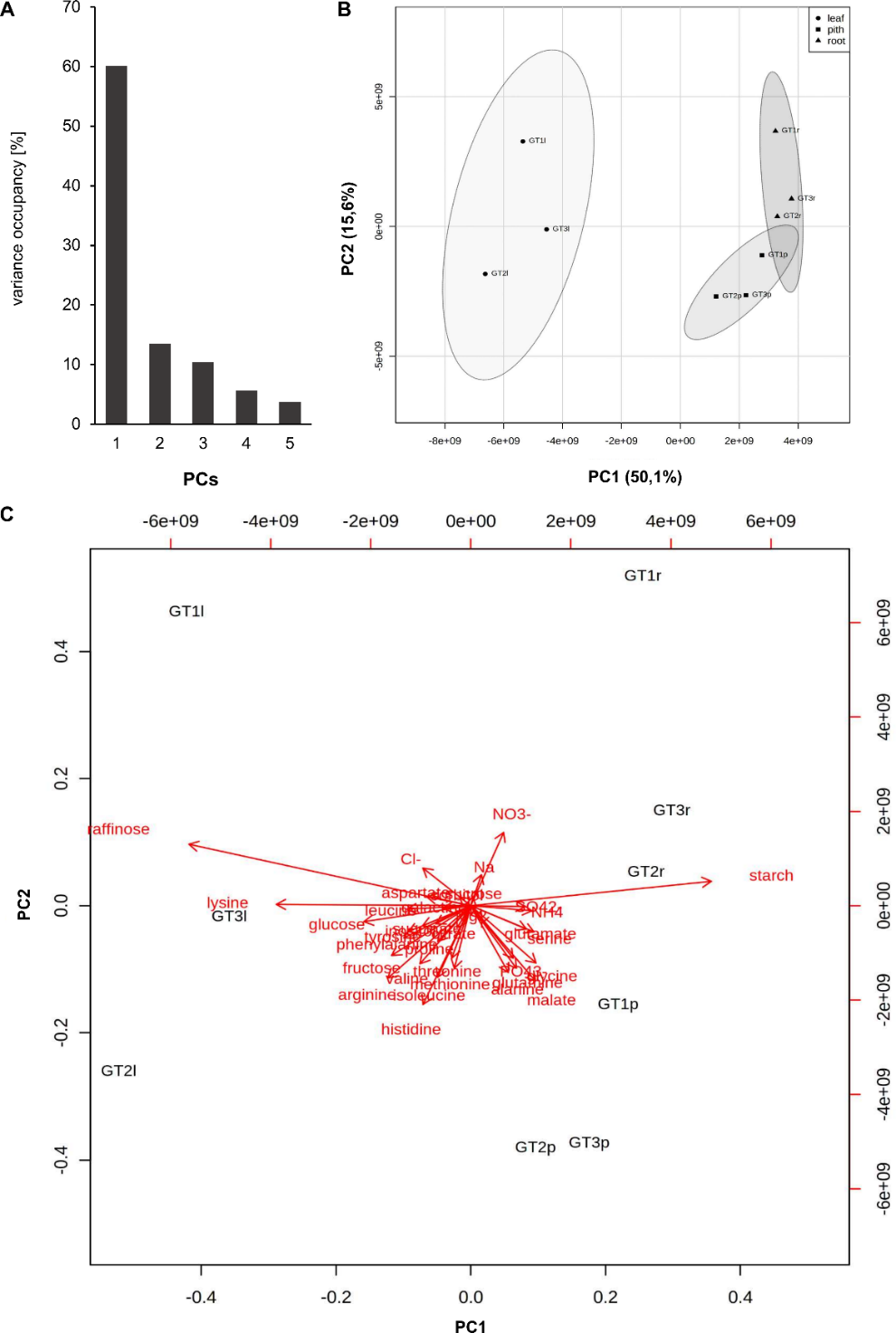


**Supplemental Figure S4: Differences in metabolite profile changes among tissues from three sugar beet cultivars upon a shift in growth temperature from 20°C to 0°C**. Differences were analyzed by Principal Component Analysis. (A) Explained variances of the first five principal components (PCs). (B) Principal component analysis (PCA) of metabolite log2 fold changes after shift in temperature from 20°C to 0°C in different sugar beet tissues. (C) The corresponding loading plot includes the names of 36 metabolites contributing to the separation of PC1 and PC2. The means of metabolite concentrations of four biological replications for each tissue and genotype measured in plants grown at 20°C and 0°C were used to calculate the log2 fold changes 20°C/0°C and used as loadings for this analysis.


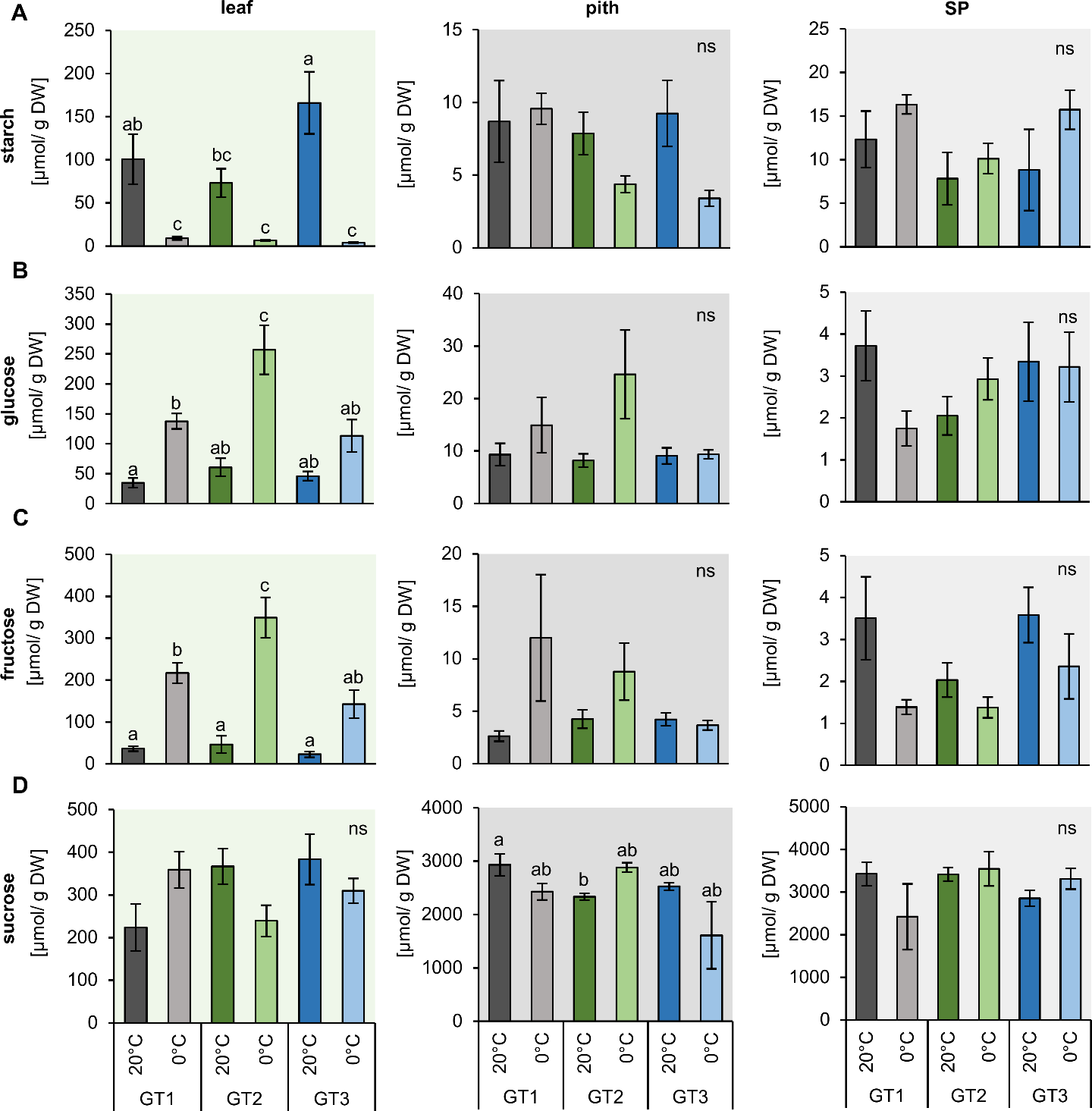


**Supplemental Figure S5: Concentrations of starch, glucose, fructose and sucrose in leaf, pith and storage root tissue under control and freezing temperatures.** Metabolites were measured in plants grown at 20°C and plants transferred to 12°C, 4°C and harvested at 0°C soil temperature. Plants were dissected in the three different tissues leaf, pith and root. Starch (A), glucose (B) fructose (C) and sucrose (D) values represent the mean of four biological replicates for each of the tested cultivars. Error bars represent the standard error of the corresponding mean. Letters indicate the same level of significance for each measured concentration, calculated via two-way ANOVA corrected with post hoc Tukey HSD test with p < 0.05.


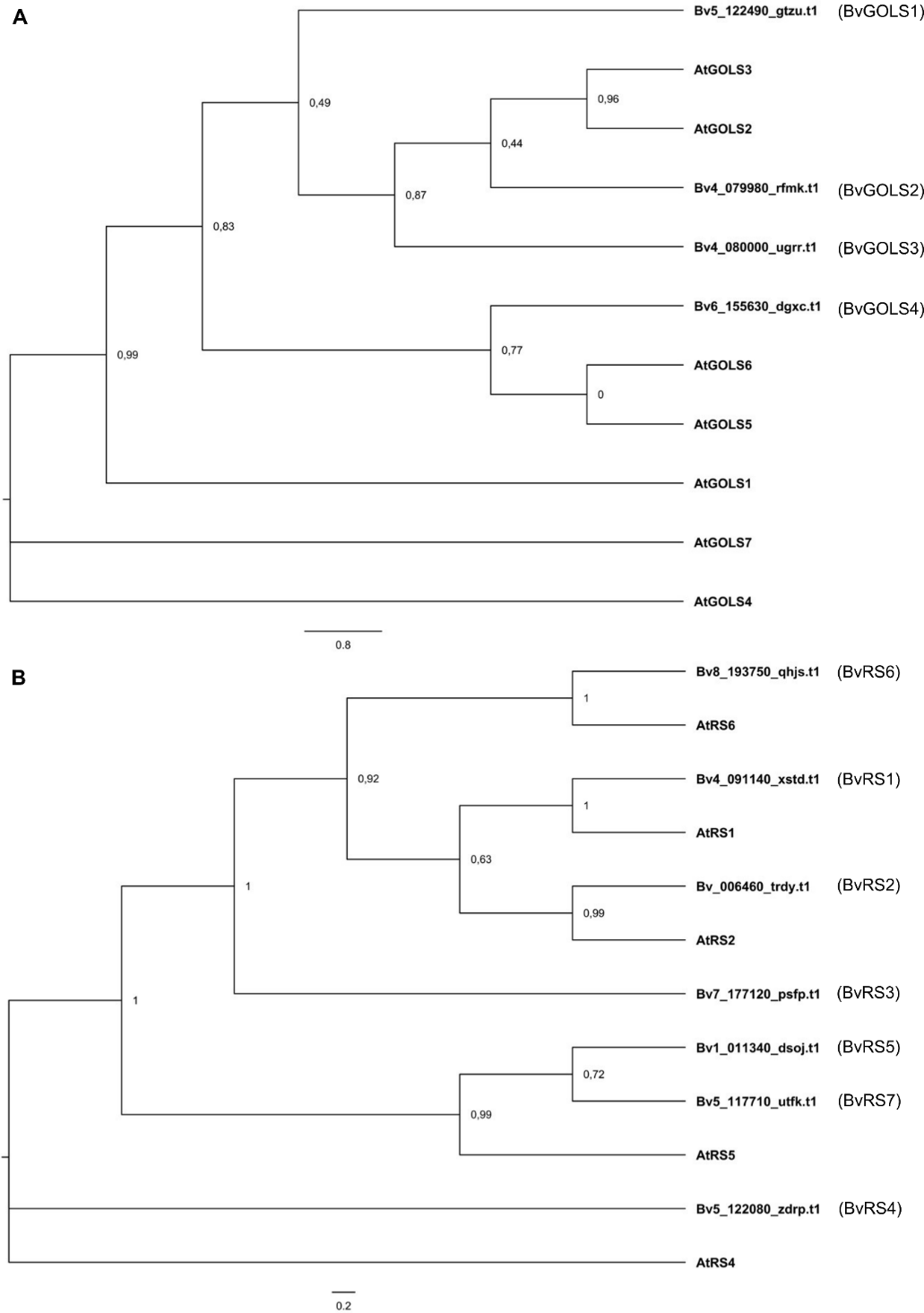


**Supplemental Figure S6: Phylogeny of *Beta vulgaris* GOLS and RS isoforms.** (A) Phylogenetic tree of galactinol synthase amino acid sequences from sugar beet and Arabidopsis thaliana. Sugar beet proteins are named according to their identifiers. Arabidopsis proteins had the following identifiers: AtGOLS1: AT2G47180; AtGOLS2: AT1G56600; AtGOLS3: AT1G09350; AtGOLS4: AT1G60470; AtGOLS5: AT5G23790; AtGOLS6: AT4G26250; AtGOLS7: AT1G60450. (B) Phylogenetic tree of raffinose synthase amino acid sequences from sugar beet and Arabidopsis thaliana. Sugar beet proteins are named according to their identifiers. Arabidopsis proteins had the following identifiers: AtRS1: AT1G55740; AtRS2: AT3G57520; AtRS4: AT4G01970; AtRS5: AT5G40390; AtRS6: AT5G20250. Phylogenetic trees were calculated using the “one-click” mode of http://www.phylogeny.fr and visualized using FigTree v1.4.4. Branch labels represent branch support values

**Supplemental Table S2: Primer used for the analysis of gene expression in *B. vulgaris* tissues.** Primer Sequences, melting temperature (Tm), as well as amplicon size and correlation coefficient (R2)used for efficiency testing (E) given for each primer used in the analysis. BvUGD1 served as a reference gene for transcript normalization


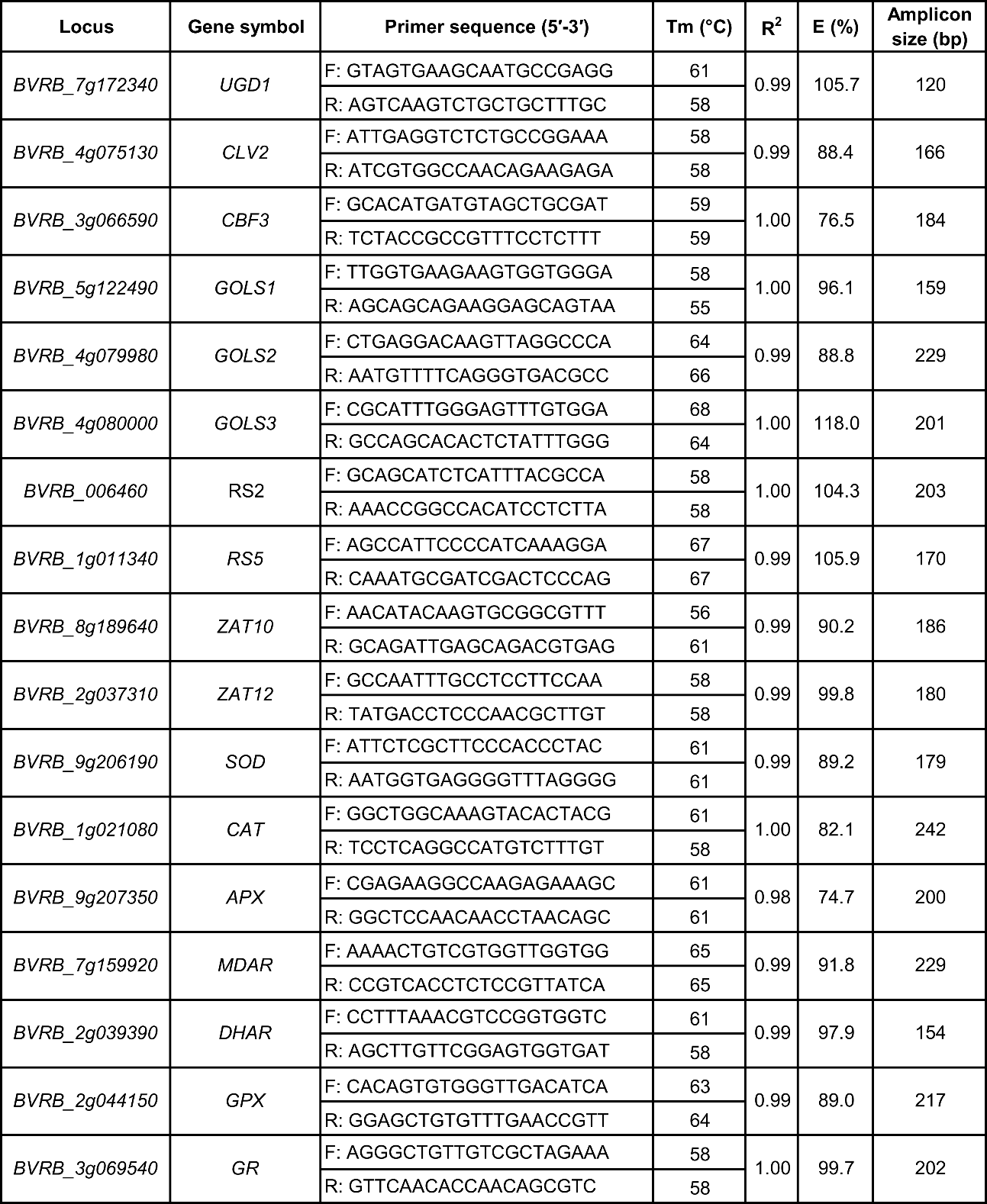


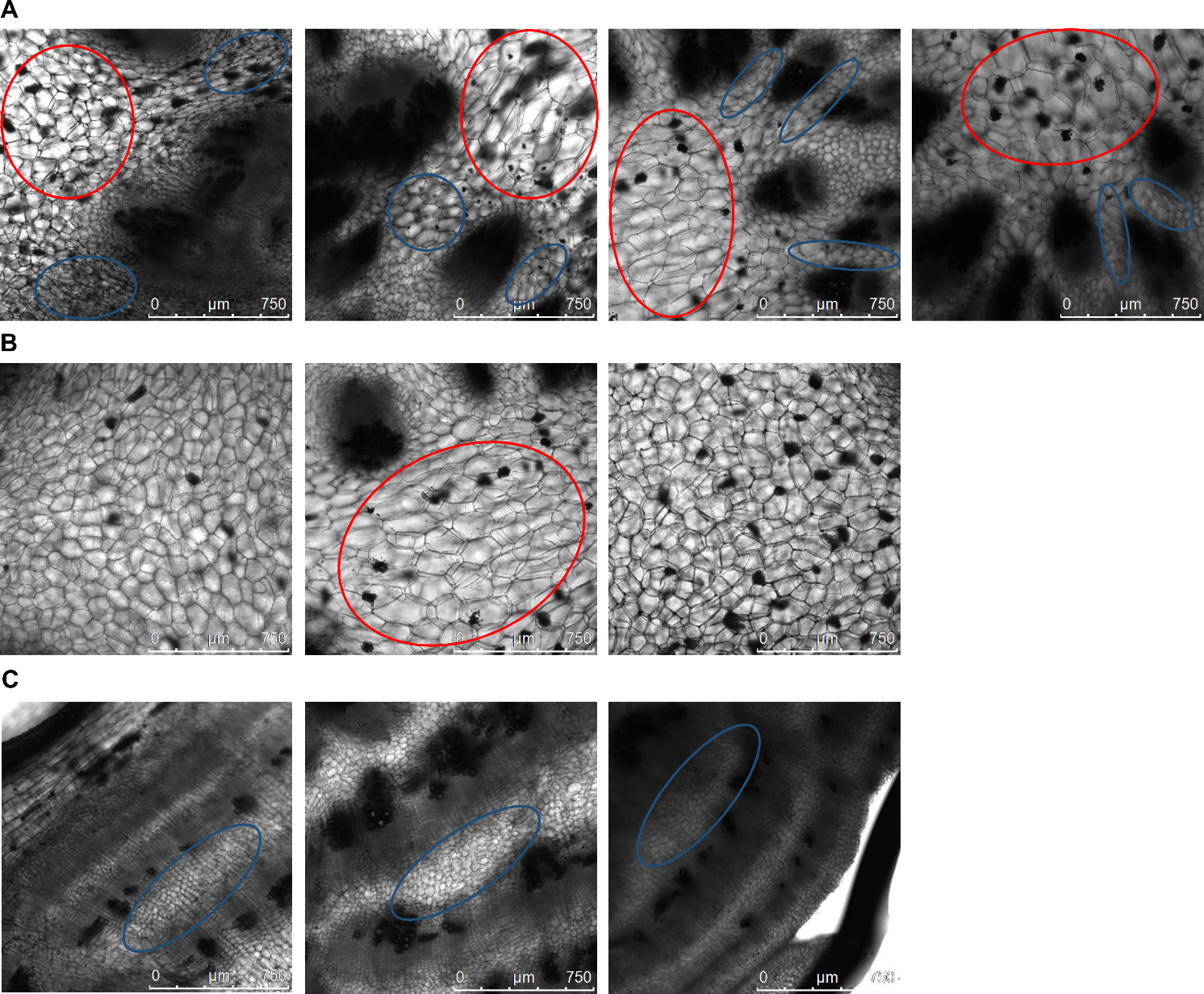


**Supplemental Figure S7: Images of pith and storage parenchyma used for measurement of cell sizes.** Pictures were taken from cross-sections of 12-week-old sugar beet taproots grown at 20°C. Red circles mark the area of the section used for measurement of pith cell sizes, blue circles for SP cell size. While pictures in panel A were used for determination of both, pith and SP cell sizes, pictures in panel B were only used for determination of pith cell sizes and in panel C for determination of SP cell size.
